# Molecular Analysis and Genomic Organization of Major DNA Satellites in Banana (*Musa* spp.)

**DOI:** 10.1371/journal.pone.0054808

**Published:** 2013-01-23

**Authors:** Jana Čížková, Eva Hřibová, Lenka Humplíková, Pavla Christelová, Pavla Suchánková, Jaroslav Doležel

**Affiliations:** Institute of Experimental Botany, Centre of the Region Haná for Biotechnological and Agricultural Research, Olomouc, Czech Republic; Ben-Gurion University, Israel

## Abstract

Satellite DNA sequences consist of tandemly arranged repetitive units up to thousands nucleotides long in head-to-tail orientation. The evolutionary processes by which satellites arise and evolve include unequal crossing over, gene conversion, transposition and extra chromosomal circular DNA formation. Large blocks of satellite DNA are often observed in heterochromatic regions of chromosomes and are a typical component of centromeric and telomeric regions. Satellite-rich loci may show specific banding patterns and facilitate chromosome identification and analysis of structural chromosome changes. Unlike many other genomes, nuclear genomes of banana (*Musa* spp.) are poor in satellite DNA and the information on this class of DNA remains limited. The banana cultivars are seed sterile clones originating mostly from natural intra-specific crosses within *M. acuminata* (A genome) and inter-specific crosses between *M. acuminata* and *M. balbisiana* (B genome). Previous studies revealed the closely related nature of the A and B genomes, including similarities in repetitive DNA. In this study we focused on two main banana DNA satellites, which were previously identified *in silico*. Their genomic organization and molecular diversity was analyzed in a set of nineteen *Musa* accessions, including representatives of A, B and S (*M. schizocarpa*) genomes and their inter-specific hybrids. The two DNA satellites showed a high level of sequence conservation within, and a high homology between *Musa* species. FISH with probes for the satellite DNA sequences, rRNA genes and a single-copy BAC clone 2G17 resulted in characteristic chromosome banding patterns in *M. acuminata* and *M. balbisiana* which may aid in determining genomic constitution in interspecific hybrids. In addition to improving the knowledge on *Musa* satellite DNA, our study increases the number of cytogenetic markers and the number of individual chromosomes, which can be identified in *Musa*.

## Introduction

A significant part of nuclear genomes in plants, including those with small genomes such as rice, *Brachypodium* and *Arabidopsis* is occupied by various types of repetitive DNA sequences [1], [2], [3]. Repetitive DNA sequences are classified based on genomic organization of repetitive units as dispersed and tandem. Dispersed repeats, represented by various classes of transposable elements encode for proteins which facilitate their replication and integration into nuclear genome [4]. Tandem repeats usually contain non-coding sequences organized in tandem arrays [5], [6], [7], [8], [9]. The arrays may be large and consist of thousands or even millions of repetitive units arranged in head-to-tail orientation [10], [11]. The processes by which satellites arise and evolve are not well understood, and unequal crossing over, gene conversion, transposition and formation of extra chromosome circular DNA (eccDNA) were implicated [12].

The analysis of repetitive DNA and satellite DNA in particular, has been hampered by the presence of multiple copies of the same or similar sequences arranged in tandem. However, the situation changed recently with the advent of new sequencing technologies, which enable identification and reconstruction of tandem organized units even from partial genome sequencing data [13], [14], [15]. On line with the analysis at DNA level, long-range organization of satellite DNA has been studied using cytogenetic methods; fluorescence *in situ* hybridization (FISH) being the most frequent. Large blocks of satellite DNA are typically observed in heterochromatic regions of chromosomes and are usually located in centromeric and telomeric regions [16], [17]. Chromosome loci rich in satellite DNA usually show specific banding pattern and this makes them useful cytogenetic markers to discriminate individual chromosomes [18], [19], [20], [21]. The presence of chromosome landmarks enables identification of individual chromosomes and is a prerequisite to study structural changes accompanying evolution and speciation and to follow chromosome behavior and transmission in interspecific hybrids [22], [23].

Bananas are a staple food and important export commodity in many countries of humid tropics. Most of banana cultivars are seed sterile diploid and triploid clones originating from natural inter- and intra-specific crosses involving wild diploid species of genus *Musa*: *M. acuminata* (A genome) and *M. balbisiana* (B genome) [24]. Both species belong to section *Eumusa* (*x* = 11); other three sections are recognized within the genus *Musa* based on chromosome number and morphology: *Rhodochlamys* (*x* = 11), *Australimusa* (*x* = 10) and *Callimusa* (*x* = 10 or *x* = 9). Nuclear genome of *Musa* is relatively small (1C×∼550–750 Mbp) [25], [26] and divided to 1–2 µm long and morphologically similar chromosomes. This makes cytogenetic studies difficult and there is no reliable method to identify all chromosomes within a karyotype and discriminate parental chromosomes in hybrids. Genomic *in situ* hybridization (GISH) has been used occasionally to identify parental chromosomes in some hybrid cultivars [27], [28]. However, the method discriminates only (peri)centromeric chromosome regions. Apart from identifying parental chromosomes, the paucity of chromosome-specific landmarks and markers hampers studies on karyotype evolution and chromosome behavior in hybrids. To date, only a few DNA sequences and probes such as rDNA, some DNA repeats and BAC clones were found useful to study the organization of plant nuclear genomes at cytogenetic level [29], [30], [31].

Recently, a variety of repetitive DNA elements including two new satellite DNA sequences were identified *in silico* from 454 sequencing data of *M. acuminata* cv. ‘Calcutta 4’ [13]. Even though most of retrotransposons were found dispersed along all banana chromosomes, some of them (a LINE element and a CRM retrotransposon) were located in (peri)centromeric regions, while the DNA satellites were identified on specific chromosome loci [13]. These results indicated a potential of these sequences as cytogenetic markers.

In this work we characterized genomic organization of two main banana DNA satellites together with other DNA sequences (a LINE element, rRNA genes, a BAC clone) in nineteen accessions of *Musa*, including inter-specific hybrids. Molecular analysis of the two DNA satellites revealed their sequence conservation within and between the accessions. The mode of their genomic distribution makes them suitable as cytogenetic markers. We show that the LINE-like element is present in centromeric regions of all nineteen accessions and can be used as centromere-specific cytogenetic marker. The present work improves the knowledge of genome structure in *Musa* and expands the number of individual chromosomes which can be identified. Differences in fluorescent labeling patterns obtained after FISH with a set of probes can be used to support determination of genomic constitution in inter-specific hybrids.

## Materials and Methods

### Plant Material and Genomic DNA Extraction

*In vitro* rooted plants of 18 *Musa* accessions were obtained from the International Transit Centre (ITC, Katholieke Universitiet, Leuven, Belgium). Five rooted plants of *M. balbisiana* clone ‘Pisang Klutuk Wulung’ were obtained from Dr. François Côte (CIRAD, Guadeloupe). The *in vitro* plants were transferred to soil and all plants were maintained in a greenhouse. Table 1 lists all accessions used in the present study.

**Table 1:** List of *Musa* accessions and their genome sizes.

Species	Subspecies	Accession name	ITC code	Genomic constitution^a^	2C nuclear DNAcontent [pg]		Mean monoploid genome size [Mbp/1C_x_]^b^	Reference^c^
					mean	± SD		
*M. acuminata*	*burmannicoides*	Calcutta4	0249	AA	1.226	0.004	600	Bartoš *et al*. [25]
	*burmannica*	Long Tavoy	0283	AA	1.238	0.01	605	*
	*zebrina*	Maia Oa	0728	AA	1.325	0.02	648	*
	*malaccensis*	DH Pahang	1511	AA	1.214	0.027	594	*
		Tuu Gia	0610	AA	1.261	0.02	617	*
*M. balbisiana*		Cameroun	0246	BB	1.121	0.003	548	Lysák *et al*. [67]
		Honduras	0247	BB	1.133	0.002	554	*
		Tani	1120	BB	1.126	0.014	551	*
		Pisang Klutuk Wulung	–	BB	1.132	0.025	554	*
*M. schizocarpa*		*Musa schizocarpa*	0560	SS	1.373	0.015	671	*
		*Musa schizocarpa*	1002	SS	1.364	0.003	667	*
Hybrids		Obino l’Ewai	0109	AAB	1.777	0.004	579	Lysák *et al*. [67]
		Maritú	0639	AAB	1.847	0.005	602	Lysák *et al*. [67]
		3 Hands Planty	1132	AAB	1.786	0.019	582	*
		Pelipita	0472	ABB	1.751	0.002	571	Lysák *et al*. [67]
		Balonkawe	0473	ABB	1.814	0.034	591	*
		Ato	0820	AS	1.294	0.015	633	*
		Tonton Kepa	0822	AS	1.318	0.021	645	*
		Umbubu	0854	AT	1.397	0.009	683	*

a Genome constitution as described in MGIS database (http://www.crop-diversity.org/banana/#AvailableITCAccessions).

b 1 pg = 0.978×10^9^ bp [34].

c The study in which DNA content was estimated.

* Estimated in the present work.

Genomic DNA was prepared from nuclei isolated from healthy young leaf tissues according to Zhang et al. [32]. Isolated nuclei were incubated with 40 mM EDTA, 0.2% SDS and 0.25 µg/µl proteinase K for 5 hours at 37°C; DNA was purified by phenol/chloroform precipitation.

### Estimation of Genome size

Nuclear genome size was estimated according to Bartoš et al. [25]. Suspensions of cell nuclei were prepared by chopping leaf tissues with a razor blade in a glass Petri dish containing 500 µl Otto I solution (0.1 M citric acid, 0.5% v/v Tween 20). Approximately 50 mg of young *Musa* leaf and 10 mg of leaf of soybean (*Glycine max* L. cv. Polanka, 2C = 2.5 pg DNA) [26] which served as internal standard were used for sample preparation. Crude homogenate was filtered through a 50 µm nylon mesh, nuclei were pelleted (300 *g*, 5 min) and resuspended in 300 µl Otto I solution. After 1 hour incubation at room temperature, 900 µl Otto II solution (0.4 M Na_2_HPO_4_ ) [33] supplemented with 50 µg/ml RNase, 50 µg/ml propidium iodide and 3 µl/ml 2-mercaptoethanol, were added. Samples were analyzed using Partec PAS flow cytometer (Partec GmbH, Münster, Germany) equipped with 488-nm argon laser. At least 5,000 nuclei were analyzed per sample. Three individuals were analyzed in each accession, and each individual was measured three times on three different days. Nuclear DNA content was calculated from individual measurements following the formula:
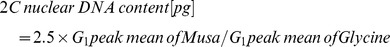



Mean nuclear DNA content was then calculated for each plant and converted to the number of base pairs considering 1 pg DNA equal to 0.978×10^9^ bp [34].

### Dot-plot

Satellites maTR_CL18 and maTR_CL33 (GenBank accessions: JX624137 and JX624137) were originally identified *in silico* after low-pass 454 sequencing genomic DNA of *M. acuminata* ‘Calcutta 4’ in our previous study [13] and their presence was confirmed in the recently published genome assembly of *M. acuminata* ‘DH Pahang’ [35]. In order to confirm tandem arrangement of both repeats prior to our experimental work, we have analyzed the sequence data using dotter [36].

### Southern Hybridization

Genomic organization of DNA satellites maTR_CL18 and maTR_CL33 [13] was analyzed in all 19 *Musa* accessions. Aliquots of genomic DNA corresponding to 1×10^7^ copies of monoploid (1Cx) nuclear genomes were digested using *Dra*I whose restriction site was identified in maTR_CL18 and maTR_CL33 sequences. Digested DNA was size-fractionated by 1.2% agarose gel electrophoresis and transferred to Hybond N+ nylon membranes (Amersham, Bath, UK). Probes for satellites CL18 and CL33 were prepared after PCR amplification from genomic DNA of *M. acuminata* ‘Calcutta 4’ using specific primers (Table 2) and labeling by biotin. Southern hybridization was done at 68°C overnight and signals were detected using BrightStar® BioDetect™ kit under manufacturer’s instructions (Ambion, Austin, USA).

**Table 2:** Primers used for PCR amplification of satellite DNA and a LINE element.

Type of repetitive DNA	DNA sequence name	Primer name	Primer sequence	PCR product length
Tandem repeat				
	CL18	CL18-1^a^	5′-ATCATGGGCCAACACTTGAT	Ladder-like pattern
			5′-TCGTGAGAGCGGGTTAGAGT	
		CL18-2^a^	5′- ATCATGGGCCAACACTTGAT	Ladder-like pattern
			5′-TGAGAGCGGGTTAGAGTTCC	
		CL18-IN1^b^	5′-CGAATGATTTGATGTCATCTCC	491 bp
			5′-AGTGTTGGCCCATCATGTTT	
		CL18-IN2^b^	5′-GCAATGTTTCAACTCATTACCAA	179 bp
			5′-GATGCTACCGGGAAAAATTG	
	CL33	CL33-1a, b	5′-AATCGATCGAACCTCGACAT	130 bp
			5′-TCCCAATAAATTTGCCTTCG	
Non-LTR retrotransposon				
	LINE element	CL1SCL8/452^a^	5′-TGAAAGCAGCTTGATTTGGA	218 bp
		CL1SCL8/452^a^	5′-CAAGGCTTGCCAACATTTTT	

a Primers used to prepare probes for FISH.

b Primers were used to prepare probes for Southern hybridization.

Serial dilutions of genomic DNA and PCR products of satellite repeats used as standards were dot-blotted onto Hybond-N+ membranes (Amersham) with the aim to estimate copy number of satellite DNA sequences in the nuclear genomes of all evaluated *Musa* accessions. PCR products of satellite DNAs labeled by biotin were used as hybridization probes. Dots of genomic DNA and standards that gave the same intensity of hybridization signals were identified after visual inspection. Copy numbers of individual probes were estimated assuming that 1 pg of genomic DNA equals 0.978×10^9^ bp [34].

### Sequencing and Sequence Data Analysis of Satellite DNA

Sequences homologous to the two satellites (in the present work termed CL18-like and CL33-like satellites) were amplified from genomic DNA of all 19 *Musa* species using specific primers (Table 2). PCR reaction mix contained 10 ng genomic DNA, 1.5 mM MgCl_2_, 0.2 mM dNTPs, 1 µM primers, 1×PCR buffer and 2 U/100 of Dynazyme™ II DNA polymerase (Finnzymes, Espoo, Finland). Amplification was performed as follows: 94°C for 5 min (1 cycle), 94°C for 50 s, 57°C for 90 s, 72°C for 50 s (35 cycles) and 72°C for 10 min (1 cycle). PCR products were purified by PCR Rapid Kit (Invitek, Berlin, Germany), ligated into pCR-XL-TOPO vector and transformed into One Shot TOP10 electrocompetent *E. coli* (Invitrogen Life Technologies, Carlsbad, USA). For each *Musa* accession, 24 to 60 cloned PCR products were sequenced. Sequencing was carried out using the BigDye Terminator v3.1 Cycle Sequencing kit (Applied Biosystems, Foster City, USA) according to the manufacturer’s instructions and run on ABI 3730×l DNA analyzer (Applied Biosystems).

Nucleotide sequences were edited using Staden Package [37] the consensus sequences of CL33-like and the two parts of CL18-like satellites were assembled using cap3 program [38]. Multiple sequence alignments were done using MAFFT program v6.717-1 (–globalpair –maxiterate 1000) [39] and graphically displayed in SeaView v4.2.1. [40]. Sequence diversity was identified using DNA Sam program [41]. Sequence logos were generated using WebLogo tool [42]. SplitsTree4 v4.1.11 [43] was used to construct cladograms based on the Jukes-Cantor, K2P and uncorrected *p*-distances. Non-parametric bootstrapping with 1000 pseudoreplicates was performed to assess the nodal support. Cladograms were drawn and edited using FigTree program (http://tree.bio.ed.ac.uk/software/figtree/).

### Chromosome Preparations

Metaphase spreads were prepared according to Doleželová et al. [29]. Actively growing root tips were pre-treated in 0.05% 8-hydroxyquinoline for 3 hrs and fixed in 3∶1 ethanol : acetic acid. Fixed roots were washed in a solution of 75 mM KCl and 7.5 mM EDTA (pH 4) and meristem tips were digested in a mixture of 2% (w/v) pectinase and 2% (w/v) cellulase in 75 mM KCl and 7.5 mM EDTA (pH 4) for 90 min at 30°C. Protoplast suspension was then filtered through a 150 µm nylon mesh and pelleted. The pellet was resuspended in 75 mM KCl and 7.5 mM EDTA (pH 4) and incubated for 5 min at room temperature. After pelleting, the protoplasts were washed three times with 70% ethanol, and 5 µl of suspension were dropped onto a slide. Shortly before drying out, 5 µl of 3∶1 fixative were added to the drop to induce protoplast bursting. Finally, the slide was rinsed in 100% ethanol and air-dried.

### Fluorescence In Situ Hybridization (FISH)

BAC clone 2G17 [30] was labeled by Dig-11-dUTP or Bio-16-dUTP Nick Translation (Roche Applied Science, Penzberg, Germany) according to manufacturer’s instructions. FISH probes for 45S rDNA and 5S rDNA were obtained by labeling *Radka*1 DNA clone (45S rDNA) and *Radka*2 DNA clone (5S rDNA) [31] with digoxigenin-11-dUTP or biotin-16-dUTP (Roche Applied Science). Both probes were labeled by PCR using M13 forward and reverse primers (Invitrogen). Banana-specific LINE element and tandem repeats maTR_CL18 and maTR_CL33 [13] were labeled with digoxigenin-11-dUTP or biotin-16-dUTP (Roche) by PCR with specific primers (Table 2).

Hybridization mixture consisting of 50% formamide, 10% dextran sulfate in 1×SSC and 1 µg/ml labeled probe was added onto slides and denatured at 80°C for 3 min. The hybridization was carried out at 37°C overnight. The sites of probe hybridization were detected using anti-digoxigenin-FITC (Roche Applied Science) and streptavidin-Cy3 (Vector Laboratories, Burlingame, USA), and the chromosomes were counterstained with DAPI. The slides were examined with Olympus AX70 fluorescence microscope and the images of DAPI, FITC and Cy-3 fluorescence were acquired separately with a cooled high-resolution black and white CCD camera. The camera was interfaced to a PC running the MicroImage software (Olympus, Tokyo, Japan).

## Results

We applied various molecular and cytogenetic approaches to study genomic organization of two major DNA satellites in *Musa*: maTR_CL18 and maTR_CL33. We first confirmed using dot-plot analysis that both repeats were indeed tandem organized (Figure S1). The molecular structure and variability of tandem organized regions was investigated after sequencing products obtained after PCR with specific primers. We then used Southern hybridization and fluorescence *in situ* hybridization to estimate the copy number of ma_TR_CL18 and maTR_CL33 and characterize distribution of the repeats on mitotic chromosomes, respectively.

### Sequence Analysis of DNA Satellites

A set of primers specific for CL18 and CL33 satellites was used to amplify these repeats from all nineteen *Musa* accessions. The PCR products were cloned and sequenced to study diversity of repeats within and between banana accessions (Tables S1, S2, S3).

Considering the length of CL18 repetitive units (2226 bp) and heterogeneity of the sequenced regions, two parts of the CL18-like repetitive unit were amplified and assembled: CL18-part1 and CL18-part2, which corresponded to 921 bp and 825 bp regions of CL18 [13]. The sequenced region represented 1728 bp (77.6%) of CL18. In most of the *Musa* accessions, sequences representing both parts of CL18 repetitive units were obtained (Tables S1, S2, S3). The exceptions were *M. acuminata* ‘Tuu Gia’ and both representatives of *M. schizocarpa*, where PCR amplification followed by sequencing did not identify sequences similar to part1 of CL18. In some other clones (*M. acuminata* ‘DH Pahang’, ‘Long Tavoy’ and ‘Maia Oa’, *M. balbisiana* ‘Honduras’ and ‘Tani’, and hybrid clones ‘Balonkawe’ (ABB), ‘Ato’ (AS) and ‘Tonton Kepa’ (AS)), relatively short sequences corresponding to part1 of CL18 were obtained. On the other hand, sequences homologous to part2 of CL18 were not obtained from hybrid clone ‘3 Hands Planty’ (AAB).

**Figure 1:**
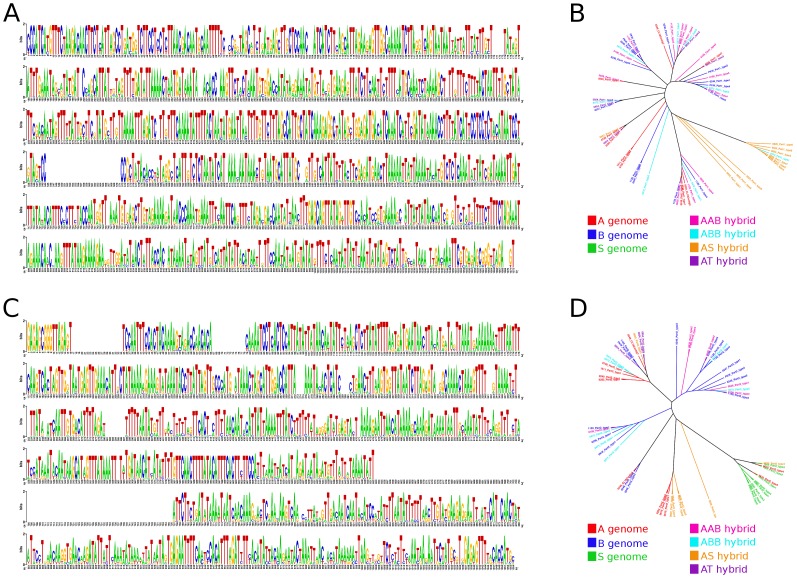
Sequence diversity of the two parts of CL18-like satellite sequences. Consensus sequences of both parts of CL18-like satellite were reconstructed from all analyzed accessions and are displayed as sequence logo (A: CL18-part1; C: CL18-part2). Neighbor-Joining trees constructed from a Jukes-Cantor distance matrix and rooted to the midpoint show diversity of reconstructed parts of CL18-like satellite units obtained from all studied species (B: CL18-part1; D: CL18-part2).

In most of the accessions, both parts of CL18-like repeat regions shared high homology to the maTR_CL18 satellite as identified in 454 data [13]. Lower sequence homology to maTR_CL18 was observed in accessions in which PCR amplification did not result in amplification of both parts of maTR_CL18 (Tables S1, S2). Sequence diversity of regions corresponding to part2 of maTR_CL18 repetitive unit was slightly lower within individual accessions as compared to part1 (Tables S1, S2). Phylogenetic analysis based on Neighbor Joining resulted in a tree in which different clades did not contain species-specific CL18-like repetitive units. Thus, we were not able to identify B genome-specific variants of CL18 suitable as B-genome specific markers (Figure 1C, D). Altogether our observations indicated that both parts homologous to CL18 satellite were highly conserved within and between the analyzed accessions (Figures 1A, B).

Sequencing PCR products obtained with primers specific for the CL33 satellite confirmed its presence in all *Musa* accessions. In several accessions (‘Maia Oa’, ‘Tuu Gia’, ‘Pelipita’, ‘3 Hands planty’, ‘Ato’ and ‘Tonton Kepa’) 188 bp repetitive units were present in addition to 134 bp repetitive units of CL33. This significant length difference was due to a 54 bp insertion/deletion. Similarly, *M. acuminata* ‘Long Tavoy’ contained a 110 bp repetitive unit of CL33.

**Figure 2:**
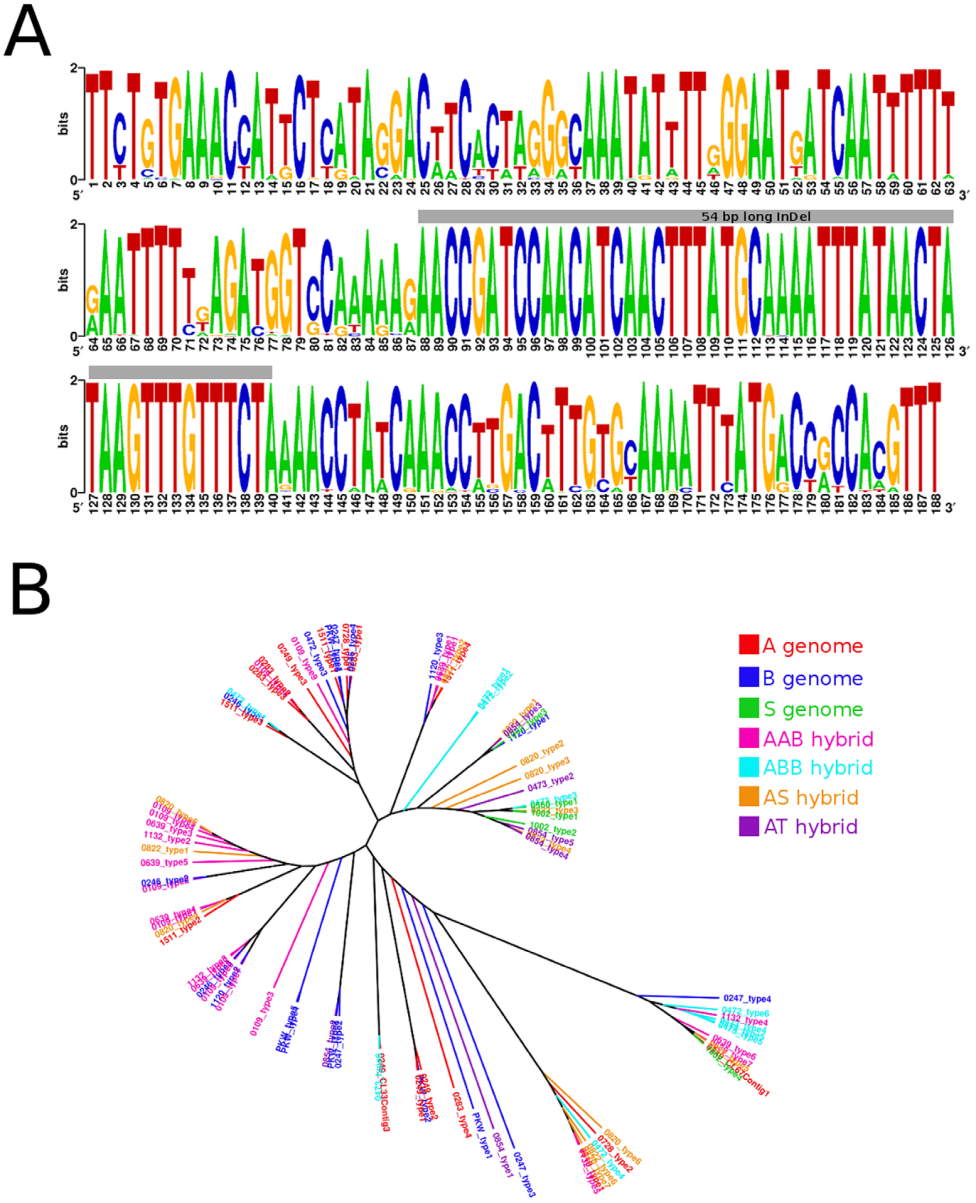
Diversity of CL33-like satellite sequence. Consensus sequence of CL33-like satellite was reconstructed from all obtained sequence units of all analyzed accessions and is displayed as sequence logo (A). Neighbor-Joining tree constructed from a Jukes-Cantor distance matrix and rooted to the midpoint shows diversity of individual types of CL33-like repetitive units obtained from all studied accessions (B).

Most of the analyzed *Musa* accessions shared high homology to maTR_CL33 satellite [13]. Lower sequence homology to maTR_CL33 was observed in both accessions of *M. schizocarpa* and its interspecific hybrid (Table S3). In general, a low sequence diversity of sequences corresponding to maTR_CL33 repetitive unit was observed. The only exceptions were revealed in ‘Balonkawe’ (ABB) and one representative of *M. balbisiana* (Table S3). The high level of homology of sequences corresponding to maTR_CL33 satellite within and between all *Musa* accessions is demonstrated in Figure 2A. Similarly to the results of CL18-like repeats, cluster analysis based on Neighbor Joining showed that there were no A or B genome-specific CL33-like satellite units in the current set of *Musa* accessions (Figure 2B).

### Nuclear DNA Content

The amount of nuclear DNA was estimated after flow cytometric analysis of propidium iodide-stained nuclei. All analyses resulted in histograms of relative DNA content with clearly defined peaks corresponding to G_1_ nuclei of *Musa* and the reference standard (*Glycine max*) with coefficient of variation ranging from 2.5% to 4.5%. 2C nuclear DNA content ranged from 1.121 pg to 1.397 pg for diploid accessions and from 1.751 pg to 1.847 pg for triploid accessions (Table 1). Among the diploid accessions, the lowest DNA content was found in *M. balbisiana* (2C = 1.121–1.133 pg), while the highest 2C DNA content of 1.397 pg was estimated in ‘Umbubu’ (AT genomic constitution).

### Southern Hybridization

**Figure 3:**
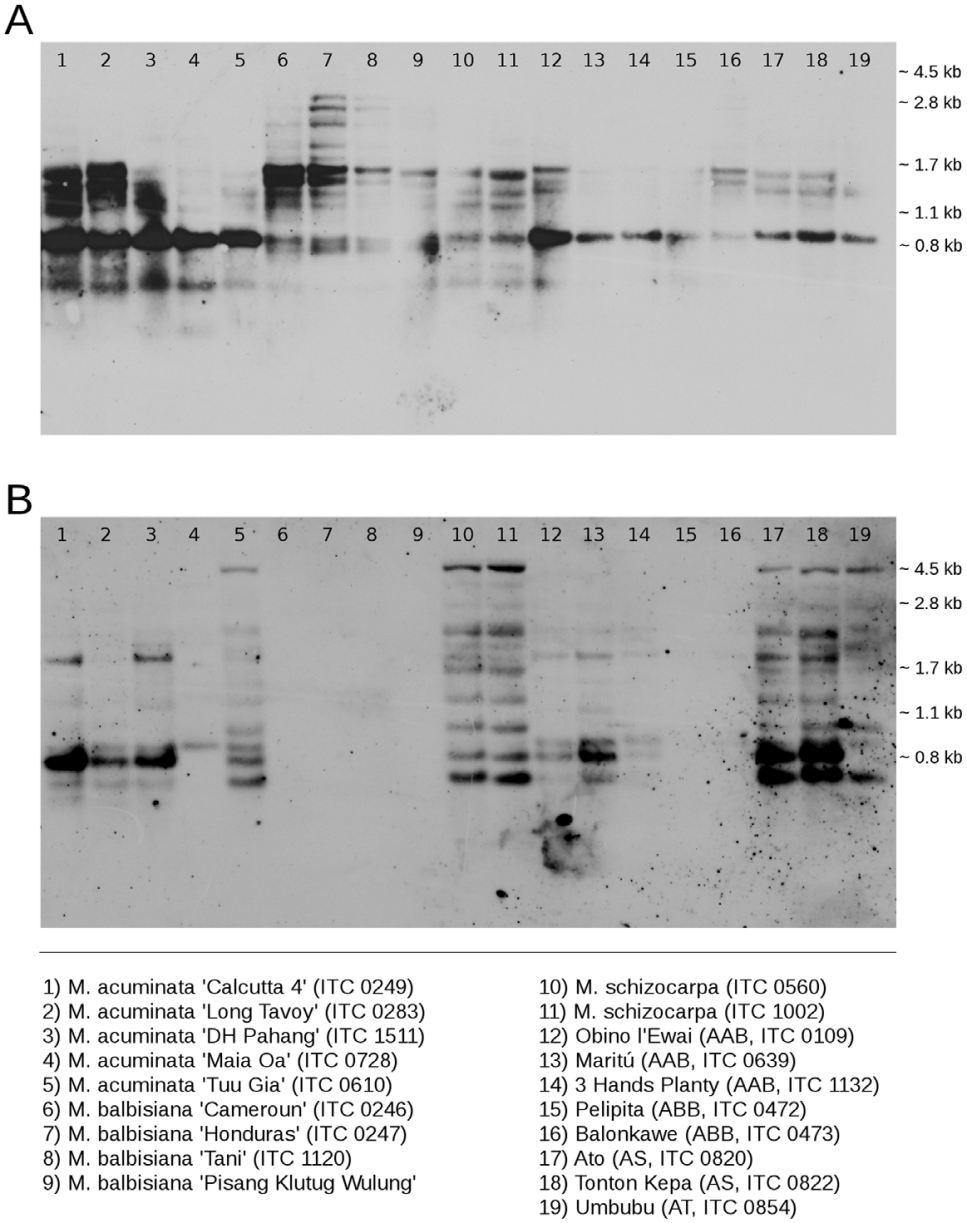
Southern hybridization of genomic DNA isolated from nineteen *Musa* accessions. Samples of genomic DNA corresponding to 1×10^7^ copies of monoploid (1C_x_) nuclear genomes were digested using *Dra*I restriction enzyme and hybridized with probes for CL18-like satellite (A) and CL33-like satellite (B) at hybridization stringency of 85%.

Southern hybridization with probes for maTR_CL18 and maTR_CL33 was carried out to investigate genomic organization of the satellites. Unfortunately, a typical pattern showing individual n-mers units was not observed, most probably because *Dra*I endonuclease did not digest all repetitive units in the analyzed accessions. Both satellites gave ladder-like pattern (Figure 3A) with various hybridization signal intensities. While the signals of maTR_CL18 repeat were visible in all *Musa* accessions, the presence maTR_CL33 repeat was not confirmed in any of the four accessions of *M. balbisiana*. Accordingly, maTR_CL33 repeat resulted in weak signals in hybrid clones with ABB genome constitution (Figure 3B).

Dot-blot analysis showed a limited variation in copy number of maTR_CL18 repeat. Its abundance ranged approximately from 1×10^3^ to 2×10^3^ per monoploid (1Cx) genome of *M. acuminata* except of *M. acuminata* ‘DH Pahang’ with 3.5×10^3^ to 5×10^3^ copies. A similar estimate (1×10^3^ to 2×10^3^) was made also in both accessions of *M. schizocarpa* and in *M. balbisiana* ‘Honduras’. The remaining *balbisiana* species contained of 3.5×10^3^ to 5×10^3^ copies of maTR_CL18 repeat. Monoploid genome of hybrid clones ‘Ato’ and ‘Tonton Kepa’ (AS) contained 1×10^3^ to 2×10^3^ copies of maTR_CL18 units, while hybrid clones ‘Umbubu’ (AT), ‘Maritú’ and ‘3 Hands Planty’ contained approximately 3×10^3^–4×10^3^ copies of the repeat. Finally, hybrid clones ‘Pelipita’ and ‘Balonkawe’ (ABB) as well as ‘Obino l’Ewai’ (AAB) comprised ∼5×10^3^ to 6.5×10^3^ copies of maTR_CL18 (Table S4).

The number of copies of maTR_CL33 repeat ranged from 2.5×10^3^ to 4×10^3^ per monoploid genome of *M. acuminata* ‘Long Tavoy’, ‘Zebrina’ and ‘Tuu Gia’ as well as in both hybrid clones ‘Ato’ and ‘Tonton Kepa’. 5.5×10^3^ to 7×10^3^ copies of maTR_CL33 repetitive units were estimated in monoploid genomes of *M. acuminata* ‘Calcutta4’ and ‘DH Pahang’ and both *schizocarpa* accessions. The same amount of maTR_CL33 (5.5×10^3^ to 7×10^3^) was revealed in hybrid clones ‘Obino l’Ewai’, ‘Maritú’ and ‘3 Hands Planty’ (AAB) as well as in ‘Umbubu’ (AT). Dot-blot analysis failed to detect presence of maTR_CL33 repeat in *M. balbisiana* species and in hybrid clones with ABB genomic constitution.

### Mapping DNA Sequences on Mitotic Chromosomes

**Figure 4:**
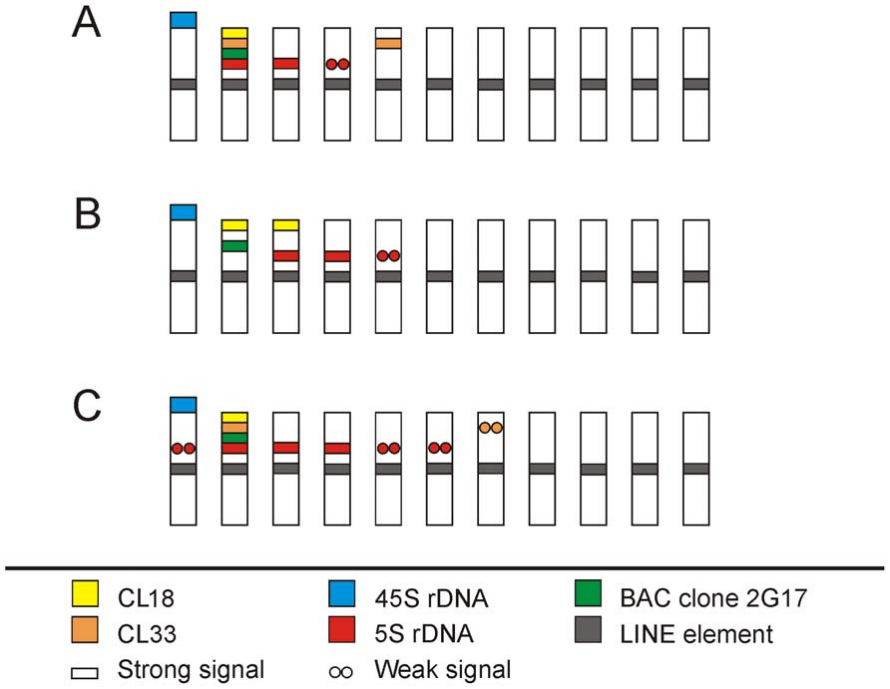
Idiograms of three diploid *Musa* accessions. (A) *M. acuminata* ‘DH Pahang’ ITC 1511; (B) *M. balbisiana* ‘Pisang Klutuk Wulung’; (C) *M. schizocarpa* ITC 0560.

In order to study chromosome organization of the two satellites and the position of their loci in relation to other cytogenetic landmarks, multicolor FISH was done in a set of 19 *Musa* species and clones with probes for single-copy BAC clone 2G17, 45S rDNA and 5S rDNA. Moreover, taking into the account the relatively small size of banana chromosomes and difficulty in identifying primary constrictions, a probe for LINE element, which was identified and characterized previously in *M. acuminata* cv. ‘Calcutta 4’ [13], was used to label putative centromeric regions. The element was found in centromeric regions of all *Musa* accessions and thus was used as a centromeric marker to aid in constructing the idiograms (Figure 4, Figure S2, Figures S3A–G).

#### 45S rRNA genes

FISH with a probe for 45S rDNA revealed its localization exclusively to secondary constriction; the only exception being a diploid hybrid clone ‘Ato’ (AS genome). Apart from this cultivar, in which additional 45S rDNA locus was observed on one of the nucleolar organizing chromosomes (Figure 3F), the number of 45S rDNA loci in mitotic metaphase plates corresponded to ploidy level (Table 3). 45S rDNA was detected in secondary constriction on one chromosome pair in diploid accessions, while three loci corresponding to 45S rDNA were identified in triploid accessions.

**Table 3:** Number of loci detected using FISH with probes for CL18 and CL33 satellites, rRNA genes and BAC clone 2G17 on mitotic metaphase plates in 19 accessions of *Musa.*

Accession name	ITC* code	Genomic constitution	Chromosome number (2n)	Number of probe signals (per metaphase plate)				
				45S rDNA	5S rDNA	2G17	CL18	CL33
Calcutta 4	0249	AA	22	2	8	2	2	4
Long Tavoy	0283	AA	22	2	4	2	2	4
Maia Oa	0728	AA	22	2	4	2	2	2
DH Pahang	1511	AA	22	2	6	2	2	4
Tuu Gia	0610	AA	22	2	4	2	2	4
Cameroun	0246	BB	22	2	4	2	3	−
Honduras	0247	BB	22	2	6	2	4	−
Tani	1120	BB	22	2	6	2	4	−
Pisang Klutuk Wulung	–	BB	22	2	6	2	4	−
*Musa schizocarpa*	0560	SS	22	2	12	2	2	4
*Musa schizocarpa*	1002	SS	22	2	12	2	2	4
Obino l’Ewai	0109	AAB	33	3	9	4	4	4
Maritú	0639	AAB	33	3	8	4	4	4
3 Hands Planty	1132	AAB	33	3	9	4	4	3
Pelipita	0472	ABB	33	3	10	3	6	1
Balonkawe	0473	ABB	33	3	10	4	6	1
Ato	0820	AS	22	2	9	2	2	4
Tonton Kepa	0822	AS	22	2	9	2	2	4
Umbubu	0854	AT	21	2	4	1	1	2

* International Transit Centre (http://www.bioversityinternational.org/research/conservation/genebanks/musa_international_transit_centre.html).

#### 5S rRNA genes

**Figure 5:**
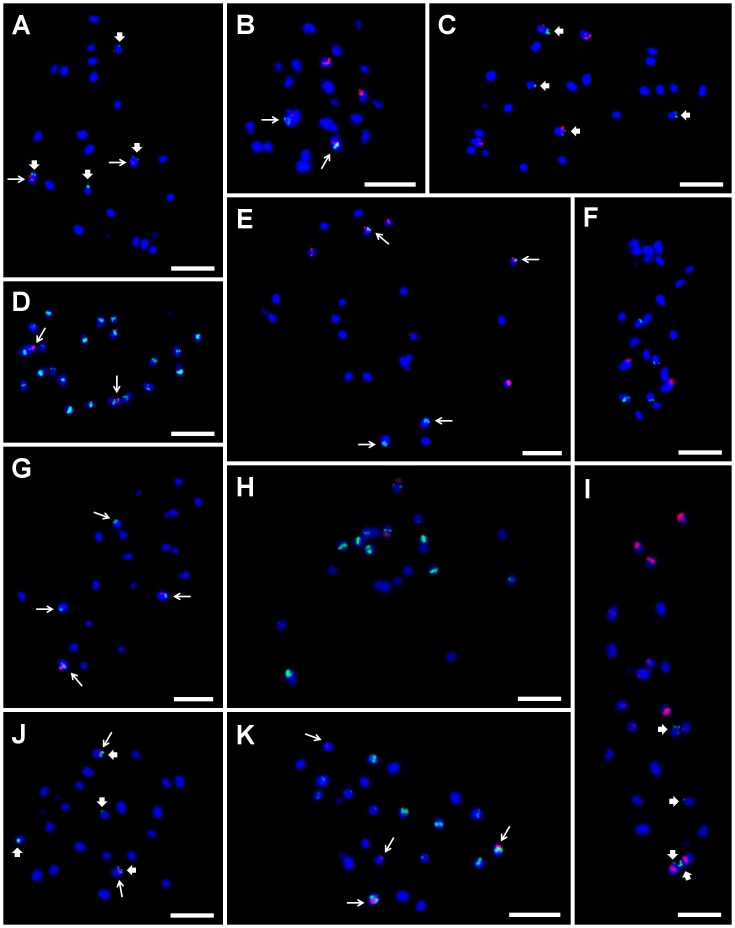
Examples of genomic distribution of DNA satellites as determined on mitotic metaphase chromosomes of diploid *Musa* accessions after FISH. Chromosomes were counterstained with DAPI (blue). Sites of CL18 and CL33 probe hybridization are marked by long and thick arrows, respectively. (A) CL18 (red) and CL33 (green) on chromosomes of ‘Long Tavoy’. (B) 5S rDNA (red) and CL18 (green) on chromosomes of ‘Maia Oa’. (C) 5S rDNA (red) and CL33 (green) on chromosomes of ‘Long Tavoy’. (D) CL18 (red) and LINE element (green) on chromosomes of ‘Tuu Gia’. (E) 5S rDNA (red) and CL18 (green) on chromosomes of ‘Pisang Klutuk Wulung’. (F) BAC clone 2G17 (red) and 5S rDNA (green) on chromosomes of ‘Cameroun’. (G) BAC clone 2G17 (red) and CL18 (green) on chromosomes of ‘Tani’. (H) 45S rDNA (red) and 5S rDNA (green) on chromosomes of *M. schizocarpa* ITC 0560. (I) 5S rDNA (red) and CL33 (green) on chromosomes of *M. schizocarpa* ITC 1002. (J) CL18 (red) and CL33 (green) on chromosomes of *M. schizocarpa* ITC 1002. (K) CL18 (red) and 5S rDNA (green) on chromosomes of *M. schizocarpa* ITC 1002. Bar = 5 µm.

Gene loci for 5S rRNA were observed mostly in distal chromosome regions in all accessions (Figures 4, 5, Figures S3A – G, Figure S4). Diploid chromosome sets of *M. acuminata* ‘Long Tavoy’, *M. acuminata* ‘TuuGia’ and *M. acuminata* ‘Maia Oa’ contained four signals corresponding to 5S rRNA genes, *M. acuminata* ‘Pahang’ contained six signals and eight loci of 5S rRNA gene clusters were detected in mitotic metaphase plates of *M. acuminata* ‘Calcutta 4’. Diploid representatives of B genome showed presence of six 5S rDNA loci with the exception of *M. balbisiana* ‘Cameroun’, which contained only four 5S rDNA clusters. Both representatives of *M. schizocarpa* (SS genome) contained six chromosome pairs bearing 5S rRNA genes (Figure 4, Figure S3C, Table 3). In triploid hybrid banana clones, eight to ten 5S rDNA loci were observed (Figures S3D, E, Table 3).

#### BAC clone 2G17

BAC clone 2G17, which was used in this work as additional cytogenetic marker, localized to subtelomeric regions on one chromosome pair in all diploid accessions with the exception of *M. balbisiana* ‘Honduras’ in which four signals were observed. Only one chromosome gave FISH signal in a diploid hybrid clone ‘Umbubu’ (AT genome), which most probably originated from a cross between *M. acuminata* and *M. textilis* (T genome). In triploid AAB and ABB clones, three or four loci containing DNA sequences homologous to BAC 2G17 were found (Figure 5, Figures S3D, E, Figure S4, Table 3).

#### Satellite repeats in diploid genomes

Satellite CL18 localized to subtelomeric regions on one pair of mitotic chromosomes in all accessions of *M. acuminata* and always co-localized with BAC clone 2G17. Satellite CL33 localized to subtelomeric regions on one chromosome pair in ‘Maia Oa’ and on two chromosome pairs of the remaining *M. acuminata* accessions. Further FISH experiments revealed that CL33 satellite co-localized with CL18 and BAC 2G17 on one chromosome pair (Figures 4, 5, Figure S3A, Table 3) in all *M. acuminata* accessions. Similar FISH patterns were observed for CL18 and CL33 in *M. schizocarpa* (Figures 4, 5, Figure S3C, Table 3).

Three *M. balbisiana* genotypes (‘Honduras’, ‘Tani’, ‘Pisang Klutuk Wulung’) contained two chromosome pairs bearing satellite CL18 in their subtelomeric regions. On one of the chromosome pairs, a probe for CL18 co-localized with BAC 2G17 and on the other co-localized with 5S rDNA (Figures 4, 5, and Figure S3B). The fourth accession of *M. balbisiana* - ‘Cameroun’ - had only three CL18 loci as a FISH signal was missing on the chromosome with 5S rDNA locus (Figure 5, and Figure S3B). CL33 gave no visible signals on metaphase chromosomes of *M. balbisiana*.

#### Satellite repeats in AAB and ABB hybrids

Out of a number of the existing interspecific hybrids with a combination of A and B genomes, three hybrid accessions with AAB genomic constitution (‘Obino l’Ewai’, ‘Maritú’ and ‘3 Hands Planty’) and two ABB hybrids (‘Pelipita’ and ‘Balonkawe’) were chosen for this study. All AAB interspecific hybrids and one ABB hybrid (‘Balonkawe’) gave signals of BAC 2G17 in subtelomeric regions of four different chromosomes. Among them, one chromosome was bearing an additional weak signal of BAC 2G17. In the second ABB hybrid ‘Pelipita’, BAC 2G17 localized in subtelomeric regions of three different metaphase chromosomes (Figures S3E, S4). 5S rDNA loci were found on eight metaphase chromosomes in the genome of ‘Maritú’, on nine chromosomes of ‘Obino l’Ewai’ and ‘3 Hands Planty’, while ‘Pelipita’ and ‘Balonkawe’ contained ten clusters corresponding to 5S rDNA.

Satellite CL18 localized in subtelomeric regions on four mitotic chromosomes in AAB hybrids; FISH with satellite CL33 resulted in three visible signals in subtelomeric regions of metaphase chromosomes of ‘3 Hands Planty’ and four signals were detected on chromosomes of ‘Obino l’Ewai’ and ‘Maritú’. Further analysis showed that two chromosomes carried both CL18 and CL33 satellite repeats. (Figures S3D, S4, Table 3). Hybrid ABB clones ‘Balonkawe’ and ‘Pelipita’ contained six signals of satellite CL18 which were detected on different chromosomes and one signal of satellite CL33. Multicolor FISH with the combination of probes corresponding to satellite repeats and 5S rRNA genes revealed one chromosome carrying CL18, CL33, 5S rDNA and BAC 2G17. Two other chromosomes exhibiting combination of FISH signals for 2G17 and CL18 satellite repeat and three additional chromosomes bearing signals for CL18 satellite and 5S rRNA genes (Figure S3E, Table 3 ).

#### Satellite repeats in AS and AT hybrids

FISH was also used to study genomic distribution of the two satellites in diploid hybrid clones ‘Ato’ and ‘Tonton Kepa’ (AS genome) and ‘Umbubu’ (AT genome). Nuclear genome of AS hybrids contained two signals of CL18 and CL33 which co-localized in subtelomeric regions on two different chromosomes, while nuclear genome of AT hybrid clone ‘Umbubu’ contained only one signal of CL18 satellite and two signals of CL33 satellite (Figure S3F, G, Table 3 ).

### Chromosome Identification

Multicolor FISH experiments with combinations of probes for 45S rDNA, 5S rDNA, banana-specific LINE-like element, DNA satellites CL18 and CL33 and a single copy BAC clone 2G17 facilitated identification of various numbers of chromosomes in diploid accessions of *Musa* (Figures 4, S3A–G, Table 3). Within the A genome representatives, three chromosomes could be distinguished in *M. acuminata* ‘Maia Oa’ and four chromosomes were identified in *M. acuminata* clones ‘Long Tavoy’ and ‘Tuu Gia’. In *M. acuminata* ‘Calcutta 4’, four chromosomes exhibited a specific hybridization pattern. Although two other chromosomes carried 5S rDNA sequences in this clone, the two chromosomes could not be discriminated from each other as the probes hybridized to similar chromosome regions. Five different chromosomes could be identified in *M. acuminata* ‘DH Pahang’.

Within the representatives of *M. balbisiana*, four chromosomes could be discriminated in ‘Cameroun’, five chromosomes were distinguished in ‘Tani’ and ‘Pisang Klutuk Wulug’ and six different chromosomes were identified in ‘Honduras’. Three different chromosomes could be recognized in the two S genome representatives. Moreover, two chromosomes carrying strong signals of 5S rDNA and other two chromosomes carrying weak signals of 5S rDNA were identified in their karyotypes. Unfortunately, the probes localized to similar chromosome regions in both cases and the chromosomes could not be differentiated from each other. Due to the lack of probes specific for A, B, S and T genomes, we were not able to identify homologous chromosomes in inter-specific hybrids.

## Discussion

Satellite repeats may undergo rapid changes in copy number and nucleotide sequence, which may result in the evolution of genus and species-specific repeats [18], [19], [20], [44], [45]. At the same time, their repetitive units are usually homogenized within a species due to concerted evolution [6], [46], [47], [48]. These features make them an attractive object for evolutionary and cytogenetic studies. Although about 45% of the *Musa* genome consists of various repetitive DNA sequences [35], satellite DNA repeats represent only ∼0.3% of the genome [13]. The presence of only two major satellite repeats in *Musa* and their low copy numbers may be related to relatively small genome and is on line with observations in some other small plant genomes [49], [50], [51].

Satellite DNA sequences are typical components of subtelomeric and centromeric chromosome regions, but may also form clusters in interstitial regions [14], [18], [52], [53], [54], [55], [56]. Our earlier studies [13], [31], [57] indicated that *Musa* does not have a typical centromeric satellite and that *Musa* centromeres are rather made of various types of retrotransposons, especially Ty3/*Gypsy*-like elements and LINE-like elements. Ty3/*Gypsy*-like elements were found in high copy numbers in centromeric regions in other plant species [58], [59], [60], [61], [62]. On the other hand, LINE elements are usually dispersed along chromosomes including their pericentromeric regions [63], [64], [65]. The present study confirmed preferential localization of the LINE element to centromeric regions in *Musa* and FISH with a probe for the LINE element appears a convenient way to label primary constrictions, which are not always easily visible on small and condensed mitotic metaphase chromosomes of *Musa*.

Our analyses revealed that nuclear genomes of all *Musa* accessions included in the present study contained the major DNA satellites CL33 and CL18. With a few exceptions, the satellites were found highly conserved (Figures 1A, B, 2A). Judging from the cluster analysis based on Neighbor Joining method, there are no A or B genome-specific satellite units in *Musa* (Figure 2B). These findings indicate that both satellites originated before the divergence of the section Eumusa (∼28 Mya) [66] and that their sequences remained conserved during speciation. Unfortunately, our observation means that the satellites are not suitable as FISH probes to discriminate A and B genome chromosomes and hence cannot help in characterizing genomic constitution in interspecific *Musa* hybrids.

One should bear in mind that our results on the length, sequence similarity to maTR_CL18 and maTR_CL33 satellites [13] as well as the nucleotide diversity may be affected by the fact that only sequences which were obtained after PCR amplification analyzed. Our results could be supported and refined after whole genome sequencing, e.g., using next generation sequencing, and reconstruction of complete repetitive elements [13].

We were not able to detect CL33 satellite by FISH on mitotic chromosomes of *M. balbisiana*, most probably because the copy number fell below the detection limit of the method. Supported by the results of Southern hybridization, these results indicate a low copy number of this satellite in the *Musa* B genome. This corresponds to lower copy number of other DNA repeats in *M. balbisiana* [31] and may be related to smaller genome size as compared to the A genome [26], [67].

Our findings extend significantly previous results of cytogenetic mapping of 45S and 5S rRNA genes and a single copy BAC clone 2G17 in *Musa* [25], [28], [29], [30], [31]. The 45S rDNA cluster localized to NORs in all accessions and the number of loci corresponded to their ploidy. The only exception was the additional locus on one satellite chromosome in diploid hybrid clone ‘Ato’ (AS genome).

5S rRNA genes are more diverse in the number of loci as well as in the genomic location. The largest variation in the number of 5S rDNA sites was observed among the accessions of *M. acuminata* where the number of loci ranged from 4 to 8 per mitotic metaphase plate. It is tempting to suggest different number of 5S loci in different subspecies of *M. acuminata*. Unfortunately, our results on a limited set of accessions do not support this idea. For example, ‘Maia Oa’ (*M. acumina* ssp. *zebrina*) and ‘Long Tavoy’ (*M. acuminata* ssp. *burmanica*) have the same number of 5S loci. On the other hand, cytogenetic observations may question the taxonomic classification of some accessions. Further work is needed to verify both options. The numbers of 5S rDNA loci in diploid accessions are on line with previous studies [25], [28], [29] except of *M. balbisiana* ‘Honduras’ where Bartoš et al [25] identified only four 5S rDNA loci. As the same genotype was analyzed in both studies (ITC 0247), the difference could be due to higher sensitivity of FISH in the present work. The highest number of 5S rDNA loci was found in *M. schizocarpa*. Twelve signals of 5S rDNA were detected in both representatives of this species, while Bartoš et al. [25] detected only six 5S rDNA sites in *M. schizocarpa* (ITC 0890). The discrepancy may be due to higher sensitivity of FISH. But as a different genotype was analyzed by Bartoš et al. [25] it is also possible that similarly to *M. acuminata*, there is a variation in the number of 5S loci in *M. schizocarpa*.

A BAC clone 2G17, which was originally identified as a single copy clone on mitotic chromosomes of *M. acuminata* ‘Calcutta 4’ [30] was localized on one chromosome pair in all diploid accessions with three exceptions. Four signals were observed on mitotic chromosomes of *M. balbisiana* ‘Honduras’. This could be a consequence of the locus duplication in this genotype. On the other hand, only three signals of CL18 were found on mitotic chromosomes of *M. balbisiana* ‘Cameroun’ which could be explained by reduction or even loss of one locus in this clone. Only one signal of CL18 was detected in metaphase plate of hybrid clone ‘Umbubu’ (AT genome) suggesting that the locus diverged between the genomes of *Eumusa* and *Australimusa* species. An alternative explanation is a reduction or loss of this region during the formation and evolution of the hybrid genome.

Due to the lack of probes specific for individual chromosomes, we were not able to identify all chromosomes within the karyotypes of eleven diploid accessions and eight inter-specific hybrids of *Musa*. Nevertheless, we have succeeded in expanding significantly the number of individual chromosomes which can be identified based on specific chromosome FISH patterns (Figure S3, Table 3). All chromosomes which could be identified unambiguously occurred in pairs of homologs in all accessions of *M. acuminata*, *M. balbisiana* and *M. schizocarpa*, indicating structural homozygosity. The only exception was found in *M. balbisiana* ‘Cameroun’ where only three loci of CL18 were found. However, our observation cannot exclude small chromosome exchanges and concerns only a subset of chromosomes. Structural heterozygosity influences chromosome behavior in meiosis, formation gametes, their genotype, distortions from expected Mendelian segregation and may cause sterility in hybrids [68]. Differences in distribution of cytogenetic landmarks within and between the diploid *Musa* species may indicate differences in chromosome structure potentially leading to aberrant meiosis and sterility in intra- and inter-specific hybrids [69], [70], [71].

The differences in FISH patterns between some chromosomes of *M. acuminata* and *M. balbisiana* provided information on the presence of some of their chromosomes in inter-specific hybrids and thus may contribute to the reconstruction of their genomic constitution. For example, triploid interspecific hybrid clones ‘Obino l’Ewai’, ‘Maritú’, and ‘3 Hands Planty’ had the expected number of CL18 loci, which is on line with the reported AAB genome constitution (Table 3). Triploid ABB clones ‘Pelipita’ and ‘Balonkawe’ were characterized by weak signals of CL33 after Southern hybridization. The presence of only one chromosome on which CL33 was detected by FISH seems to support the ABB genomic constitution with only one A genome (or its part) present.

Our results indicate that NOR-bearing satellite chromosomes are maintained in all hybrid clones we have analyzed. On the other hand, variation in the number of 5S rDNA loci was observed among the triploid hybrids (Table 3). Specific numbers of 5S rDNA loci in A and B genotypes could aid in identification of the origin of hybrids. However, this would be only possible if genomic organization of 5S loci is known in a larger set of *M. acuminata* and *M. balbisiana* accessions covering the existing diversity.

FISH analysis on interspecific hybrids revealed deviations in the number of loci of BAC clone 2G17 and satellite DNA sequences from the expected numbers based on reported genomic constitution. Instead of the expected three hybridization sites of BAC 2G17, four loci were identified in three out of four triploid hybrids. Based on our observations in diploids, there should be a maximum of five CL18 loci in a triploid hybrid. However, both ABB hybrids had six loci (Table 3). As we analyzed a limited set of diploid genotypes, we cannot exclude that other diploid genotypes have different number of CL18 loci and that the hybrids originated from them. The discrepancies between the expected and observed number of loci could be also due to backcrossing of primary hybrids to one of the parental species and a loss or gain of some chromosome types. Thus, our results may provide a support to the backcross hypothesis of De Langhe et al. [72]. Clearly, further analysis is needed to clarify the issue as tandem organized sequences can increase or decrease their copy number during the interspecific hybridization and polyploidization or can be removed completely from a hybrid genome [19], [73], [74]. Consequently they should be used with caution when determining genomic constitution in interspecific *Musa* hybrids.

## Supporting Information

Figure S1 **(A) Dot-plot comparison of maTR_CL18 which was identified in 454 sequence data of** ***M. acuminata*** **‘Calcutta 4’** [**13**] **and CL18-like repeat which was identified in whole genome sequence of** ***M. acuminata*** **‘DH Pahang’** [**35**] **.** Both repetitive units are more than 2.2 kb long and are organized in tandem arrays. (B) Dot-plot comparison of maTR_CL33 which was identified in 454 data of *M. acuminata* ‘Calcutta 4’ [13] and CL33-like repeat which was identified in whole genome sequence of *M. acuminata* ‘DH Pahang’ [35]. Both repetitive units are 134 bp long and are organized in tandem arrays. Sequence similarities are represented by dots and diagonal lines (A, B).
(TIFF)

Figure S2 **Examples of genomic distribution of satellite DNA as determined on mitotic metaphase chromosomes of** ***Musa*** **after FISH with labeled probes for CL18 (red, labeled by arrows) and banana LINE element (green).** Chromosomes were counterstained with DAPI (blue). (A) ‘Maia Oa’. (B) ‘Long Tavoy’. (C) ‘Tani’. (D) *M. schizocarpa* ITC 0560. (E) ‘Pelipita’. (F) *M. schizocarpa* ITC 1002. (G) ‘Obino l’Ewai’. (H) ‘Maritú’. (I) ‘Ato’. (J) ‘Tonton Kepa’. (K) ‘Umbubu’. Bar = 5 µm.
(TIFF)

Figure S3 **Idiograms of diploid (A – C) and hybrid (D – G)** ***Musa*** **accessions.** (A) *M. acuminata*, (B) *M. balbisiana*, (C) *M. schizocarpa*, (D) Hybrids with AAB genomics constitution, (E) Hybrids with ABB genomic constitution, (F) Hybrids with AS genomic constitution, (G) Hybrids with AT genomic constitution. No attempt was made to identify homologs in the hybrids (D – G) and all chromosomes are shown. The chromosome sizes are only indicative.
(PDF)

Figure S4 **Examples of genomic distribution of satellite DNA as determined on mitotic metaphase chromosomes of interspecific hybrids after FISH.** Chromosomes were counterstained with DAPI (blue). Sites of CL18 and CL33 probe hybridization are marked by long and thick arrows, respectively. (A) CL18 (red) and CL33 (green) on chromosomes of ‘Maritú’. (B) CL18 (red) and 5S rDNA (green) on chromosomes of ‘Maritú’. (C) 5S rDNA (red) and CL33 (green) on chromosomes of ‘Maritú’. (D) CL18 (red) and BAC clone 2G17 (green) on chromosomes of ‘Pelipita’. (E) CL18 (red) and 5S rDNA on chromosomes of ‘Pelipita’. (F) CL18 (red) and CL33 (green) on chromosomes of ‘Ato’. (G) CL18 (red) and 5S rDNA (green) on chromosomes of ‘Tonton Kepa’. (H) CL18 (red) and CL33 (green) on chromosomes of ‘Umbubu’. (I) 5S rDNA (red) and CL33 (green) on chromosomes of ‘Ato’. (J) CL18 (red) and CL33 (green) on chromosomes of ‘Tonton Kepa’. (K) 5S rDNA (red) and CL18 (green) on chromosomes of ‘Umbubu’. Bar = 5 µm.
(TIFF)

Table S1 **Basic characteristics and nucleotide diversity of part1 of CL18-like repeats.**
(DOC)

Table S2 **Basic characteristics and nucleotide diversity of part2 of CL18-like repeats.**
(DOC)

Table S3 **Basic characteristics and nucleotide diversity of CL33-like repeats.**
(DOC)

Table S4 **Copy numbers of maTR_CL18 and maTR_CL33 satellites.**
(DOC)

## References

[1] Bennetzen JL, Kellog EA (1997) Do plants have a one-way ticket to genomic obesity? Plant Cell 9: 1509–1514. 12237393 10.1105/tpc.9.9.1509 PMC157029

[2] Ingham LD, Hanna WW, Baier JW, Hannah LC (1993) Origin of the main class of repetitive DNA within selected *Pennisetum* species. Mol Gen Genet 238: 350–356. 8492802 10.1007/BF00291993

[3] Shapiro JA, von Sternberg R (2005) Why repetitive DNA is essential to genome function. Biol Rev 80: 227–250. 15921050 10.1017/s1464793104006657

[4] Kubis S, Schmidt T, Heslop-Harrison JS (1998) Repetitive DNA elements as a major component of plant genomes. Ann Bot 82: 45–55.

[5] Charlesworth B, Sniegowski PE, Stephan W (1994) The evolutionary dynamics of repetitive DNA in eukaryotes. Nature 37: 215–220. 10.1038/371215a0 8078581

[6] Elder JF Jr, Turner BJ (1995) Concerted evolution of repetitive DNA sequences in eukaryotes. Q Rev Biol 70: 297–320. 7568673 10.1086/419073

[7] Cheng Z, Dong F, Langdon T, Ouyang S, Buell CR, et al (2002) Functional rice centromeres are marked by a satellite repeat and a centromere-specific retrotransposon. Plant Cell 14: 1691–1704. 12172016 10.1105/tpc.003079 PMC151459

[8] Ellegren H (2004) Microsatellites: simple sequences with complex evolution. Nat Rev Genet 5: 435–445. 15153996 10.1038/nrg1348

[9] Tek AL, Jiang J (2004) The centromere regions of potato chromosomes contain megabase-sized tandem arrays of telomere-similar sequence. Chromosoma 113: 77–83. 15258808 10.1007/s00412-004-0297-1

[10] Macas J, Požárková D, Navrátilová A, Nouzová M, Neumann P (2000) Two new families of tandem repeats isolated from genus *Vicia* using genomic self-priming PCR. Mol Gen Genet 263: 741–751. 10905342 10.1007/s004380000245

[11] Willard HF (1991) Evolution of alpha satellite. Curr Opin Genet Dev 1: 509–514. 1822282 10.1016/s0959-437x(05)80200-x

[12] Navrátilová A, Koblížková A, Macas J (2008) Survey of extrachromosomal circular DNA derived from plant satellite repeats. BMC Plant Biol 8: 90. 18721471 10.1186/1471-2229-8-90 PMC2543021

[13] Hřibová E, Neumann P, Matsumoto T, Roux N, Macas J, et al (2010) Repetitive part of the banana (*Musa acuminata*) genome investigated by low-depth 454 sequencing. BMC Plant Biol 10: 204. 20846365 10.1186/1471-2229-10-204 PMC2956553

[14] Macas J, Neumann P, Navrátilová A (2007) Repetitive DNA in the pea (*Pisum sativum* L.) genome: comprehensive characterization using 454 sequencing and comparison to soybean and *Medicago truncatula* . BMC Genomics 8: 427. 18031571 10.1186/1471-2164-8-427 PMC2206039

[15] Macas J, Kejnovský E, Neumann P, Novák P, Koblížková A, et al (2011) Next generation sequencing-based analysis of repetitive DNA in the model dioecious plant *Silene latifolia* . PLoS One 6: e27335. 22096552 10.1371/journal.pone.0027335 PMC3212565

[16] Schmidt T, Heslop-Harrison JS (1998) Genomes, genes and junk: the large-scale organization of plant chromosomes. Trends Plant Sci 3: 195–199.

[17] Zatloukalová P, Hřibová E, Kubaláková M, Suchánková P, Šimková H, et al (2011) Integration of genetic and physical maps of chickpea (*Cicer arietinum* L.) genome using flow-sorted chromosomes. Chromosome Res 19: 729–739. 21947955 10.1007/s10577-011-9235-2

[18] Han YH, Zhang ZH, Liu JH, Lu JY, Huang SW, et al (2008) Distribution of the tandem repeat sequences and karyotyping in cucumber (*Cucumis sativus* L.) by fluorescence in situ hybridization. Cytogenet Genome Res 122: 80–88. 18931490 10.1159/000151320

[19] Macas J, Navrátilová A, Koblížková A (2006) Sequence homogenization and chromosomal localization of VicTR-B satellites differ between closely related *Vicia* species. Chromosoma 115: 437–447. 16788823 10.1007/s00412-006-0070-8

[20] Navrátilová A, Neumann P, Macas J (2003) Karyotype analysis of four *Vicia* species using *in situ* hybridization with repetitive sequences. Ann Bot 91: 921–926. 12770847 10.1093/aob/mcg099 PMC4242401

[21] Sharma S, Raina SN (2005) Organization and evolution of highly repeated satellite DNA in plant chromosomes. Cytogenet Genome Res. 109: 15–26. 10.1159/000082377 15753554

[22] Kopecký D, Havránková M, Loureiro J, Castro S, Lukaszewski AJ, et al (2010) Physical distribution of homoeologous recombination in individual chromosomes of *Festuca pratensis* in *Lolium multiflorum*. Cytogenet Genome Res. 120: 370–383. 10.1159/000313379 20501979

[23] Kopecký D, Lukaszewski AJ, Doležel J (2008) Cytogenetics of Festulolium (*Festuca*×*Lolium* hybrids). Cytogenet Genome Res. 120: 370–383. 10.1159/000121086 18504366

[24] Simmonds NW, Shepherd K (1955) The taxonomy and origins of the cultivated bananas. J Linn Soc Bot 55: 302–312.

[25] Bartoš J, Alkhimova O, Doleželová M, De Langhe E, Doležel J (2005) Nuclear genome size and genomic distribution of ribosomal DNA in *Musa* and *Ensete* (Musaceae): taxonomic implications. Cytogenet Genome Res 109: 50–57. 15753558 10.1159/000082381

[26] Doležel J, Doleželová M, Novák FJ (1994) Flow cytometric estimation of nuclear DNA amount in diploid bananas (*Musa acuminata* and *Musa balbisiana*). Biol Plant 36: 351–357.

[27] D’Hont A, Paget-Goy A, Escoute J, Carreel F (2000) The interspecific genome structure of cultivated banana, *Musa* spp. revealed by genomic DNA *in situ* hybridization. Theor Appl Genet 100: 177–183.

[28] Osuji JO, Harrison G, Crouch J, Heslop-Harrison JS (1997) Identification of the genomic constitution of *Musa* L. lines (bananas, plantains and hybrids) using molecular cytogenetics. Ann Bot 80: 787–793.

[29] Doleželová M, Valárik M, Swennen R, Horry JP, Doležel J (1998) Physical mapping of the 18S–25S and 5S ribosomal RNA genes in diploid bananas. Biol Plant 41: 497–505.

[30] Hřibová E, Doleželová M, Doležel J (2008) Localization of BAC clones on mitotic chromosomes of *Musa acuminata* using fluorescence *in situ* hybridization. Biol Plant 52: 445–452.

[31] Valárik M, Šimková H, Hřibová E, Šafář J, Doleželová M, et al (2002) Isolation, characterization and chromosome localization of repetitive DNA sequences in bananas (*Musa* spp.). Chromosome Res 10: 89–100. 11993938 10.1023/a:1014945730035

[32] Zhang HB, Zhao X, Ding X, Paterson AH, Wing RA (1995) Preparation of megabase-size DNA from plant nuclei. Plant J 7: 175–184.

[33] Otto F (1990) DAPI staining of fixed cells for high-resolution flow cytometry of nuclear DNA. In: Crissman HA, Darzynkiewicz Z, editors. Methods in Cell Biology. Academic Press, New York, Vol. 33. Pp. 105–110. 10.1016/s0091-679x(08)60516-6 1707478

[34] Doležel J, Bartoš J, Voglmayr H, Greilhuber J (2003) Nuclear DNA content and genome size of trout and human. Cytometry A 51: 127–128. 12541287 10.1002/cyto.a.10013

[35] D’Hont A, Denoeud F, Aury JM, Baurens FC, Carreel F, et al (2012) The banana (*Musa acuminata*) genome and the evolution of monocotyledonous plants. Nature 488: 213–217. 22801500 10.1038/nature11241

[36] Sonnhammer ELL, Durbin R (1995) A dot-matrix program with dynamic threshold control suited for genomic DNA and protein sequence analysis. Gene 167: GC1–GC10. 8566757 10.1016/0378-1119(95)00714-8

[37] Staden R (1996) The Staden sequence analysis package. Mol Biotechnol 5: 233–241. 8837029 10.1007/BF02900361

[38] Huan X, Madan A (1999) CAP3: A DNA sequence assembly program. Genome Res 9: 868–877. 10508846 10.1101/gr.9.9.868 PMC310812

[39] Katoh K, Kuma K, Toh H, Miyata T (2005) MAFFT version 5: improvement in accuracy of multiple sequence alignment. Nucleic Acids Res 33: 511–518. 15661851 10.1093/nar/gki198 PMC548345

[40] Galtier N, Gouy M, Gautier C (1996) SeaView and Phylo_win, two graphic tools for sequence alignment and molecular phylogeny. Comput Appl Biosci 12: 543–548. 9021275 10.1093/bioinformatics/12.6.543

[41] Eckert AJ, Liechty JD, Tearse BR, Pande B, Neale DB (2010) DnaSAM: Software to perform neutrality testing for large datasets with complex null models. Mol Ecol Resour 10: 542–545. 21565054 10.1111/j.1755-0998.2009.02768.x

[42] Crooks GE, Hon G, Chandonia JM, Brenner SE (2004) WebLogo: a sequence logo generator. Genome Res 14: 1188–1190. 15173120 10.1101/gr.849004 PMC419797

[43] Huson DH, Bryant D (2006) Application of phylogenetic networks in evolutionary studies. Mol Biol Evol 23: 254–267. 16221896 10.1093/molbev/msj030

[44] Dechyeva D, Gindullis F, Schmidt T (2003) Divergence of satellite DNA and interspersion of dispersed repeats in the genome of the wild beet *Beta procumbens* . Chromosome Res 11: 3–21. 12675302 10.1023/a:1022005514470

[45] King K, Jobst J, Hemleben V (1995) Differential homogenization and amplification of two satellite DNAs in the genus *Cucurbita* (Cucurbitaceae). J Mol Evol 41: 996–1005. 8587146 10.1007/BF00173181

[46] Dover GA, Strachan T, Coen ES (1982) Molecular drive. Science 218: 1069. 10.1126/science.7146895 7146894

[47] Baldwin BG, Sanderson MJ, Porter MJ, Wojciechowski MF, Campbell CS, et al (1995) The ITS region of nuclear ribosomal DNA: a valuable source of evidence on angiosperm phylogeny. Ann Mo Bot Gard 82: 247–277.

[48] Dover G (2002) Molecular drive. Trends Genet 18: 587–589. 12414190 10.1016/s0168-9525(02)02789-0

[49] Arabidopsis Genome Initiative (2000) Analysis of the genome sequence of the flowering plant *Arabidopsis thaliana* . Nature 408: 796–815. 11130711 10.1038/35048692

[50] International Brachypodium Initiative (2010) Genome sequencing and analysis of the model grass *Brachypodium distachyon* . Nature 463: 763–768. 20148030 10.1038/nature08747

[51] International Rice Genome Sequencing Project (2005) The map-based sequence of the rice genome. Nature 436: 793–800. 16100779 10.1038/nature03895

[52] Galasso I, Schmidt T, Pignone D, Heslop-Harrison JS (1995) The molecular cytogenetics of *Vigna unguiculata* (L.) Walp: the physical organization and characterization of 18S-5.8S-25S rRNA genes, 5S rRNA genes, telomere-like sequences, and a family of centromeric repetitive DNA sequences. Theor Appl Genet 91: 928–935. 24169979 10.1007/BF00223902

[53] Houben A, Schubert I (2003) DNA and proteins of plant centromeres. Curr Opin Plant Biol 6: 554–560. 14611953 10.1016/j.pbi.2003.09.007

[54] Jiang J, Birchler JA, Parrott WA, Dawe RK (2003) A molecular view of plant centromeres. Trends Plant Sci 8: 570–574. 14659705 10.1016/j.tplants.2003.10.011

[55] Macas J, Neumann P, Novák P, Jiang J (2010) Global sequence characterization of rice centromeric satellite based on oligomer frequency analysis in large-scale sequencing data. Bioinformatics 26: 2101–2108. 20616383 10.1093/bioinformatics/btq343

[56] Torres GA, Gong Z, Iovene M, Hirsch CD, Buell CR, et al (2011) Organization and evolution of subtelomeric satellite repeats in the potato genome. G3: Genes, Genomes, Genetics 1: 85–92. 22384321 10.1534/g3.111.000125 PMC3276127

[57] Hřibová E, Doleželová M, Town CD, Macas J, Doležel J (2007) Isolation and characterization of the highly repeated fraction of the banana genome. Cytogenet Genome Res 119: 268–274. 18253041 10.1159/000112073

[58] Bao W, Zhang W, Yang Q, Zhang Y, Han B, et al (2006) Diversity of centromeric repeats in two closely related wild rice species, *Oryza officinalis* and *Oryza rhizomatis* . Mol Genet Genomics 275: 421–430. 16463049 10.1007/s00438-006-0103-2

[59] Gindullis F, Desel C, Galasso I, Schmidt T (2001) The large-scale organization of the centromeric region in *Beta* species. Genome Res 11: 253–265. 11157788 10.1101/gr.162301 PMC311043

[60] Kumekawa N, Ohmido N, Fukui K, Ohtsubo E, Ohtsubo H (2001) A new gypsy-type retrotransposon, RIRE7: preferential insertion into the tandem repeat sequence TrsD in pericentromeric heterochromatin regions of rice chromosomes. Mol Genet Genomics 265: 480–488. 11405631 10.1007/s004380000436

[61] Neumann P, Navrátilová A, Koblížková A, Kejnovský E, Hřibová E, et al (2011) Plant centromeric retrotransposons: a structural and cytogenetic perspective. Mob DNA 2: 4. 21371312 10.1186/1759-8753-2-4 PMC3059260

[62] Presting GG, Malysheva L, Fuchs J, Schubert I (1998) A TY3/GYPSY retrotransposon-like sequence localizes to the centromeric regions of cereal chromosomes. Plant J 16: 721–728. 10069078 10.1046/j.1365-313x.1998.00341.x

[63] Kubis SE, Heslop-Harrison JS, Desel C, Schmidt T (1998) The genomic organization of non-LTR retrotransposons (LINEs) from three *Beta* species and five other angiosperms. Plant Mol Biol 36: 821–31. 9520275 10.1023/a:1005973932556

[64] Kumar A, Bennetzen JL (1999) Plant retrotransposons. Annu Rev Genet 33: 479–532. 10690416 10.1146/annurev.genet.33.1.479

[65] Schmidt T, Kubis S, Heslop-Harrison JS (1995) Analysis and chromosomal localisation of retrotransposons in sugar beet (*Beta vulgaris*) LINES and Ty1-copia- like elements as major components of the genome. Chromosome Res 3: 335–45. 7551548 10.1007/BF00710014

[66] Christelová P, Valárik M, Hřibová E, De Langhe E, Doležel J (2011) A multi gene sequence- based phylogeny of the Musaceae (banana) family. BMC Evol Biol 11: 103. 21496296 10.1186/1471-2148-11-103 PMC3102628

[67] Lysák MA, Doleželová M, Horry JP, Swennen R, Doležel J (1999) Flow cytometric analysis of nuclear DNA content in *Musa* . Theor Appl Genet 98: 1344–1350.

[68] Cai X, Xu SS (2007) Meiosis-driven genome variation in plants. Curr Genomics 8: 151–161. 18645601 10.2174/138920207780833847 PMC2435351

[69] Dodds KS, Simmonds NW (1948) Sterility and parthenocarpy in diploid hybrids of *Musa* . Heredity 2: 101–117. 18863987 10.1038/hdy.1948.6

[70] Shepherd K (1999) Cytogenetics of the genus *Musa*. IPGRI-INIBAP. Pp.160.

[71] Wilson GB (1946) Cytological studies in the Musae. II. Meiosis in some diploid clones. Genetics 31: 475–482. 20998136

[72] De Langhe E, Hřibová E, Carpentier S, Doležel J, Swennen R (2010) Did backcrossing contribute to the origin of hybrid edible bananas? Ann Bot 106: 849–857. 20858591 10.1093/aob/mcq187 PMC2990659

[73] Tek AL, Song JQ, Macas J, Jiang J (2005) Sobo, a recently amplified satellite repeat of potato, and its implications for the origin of tandemly repeated sequences. Genetics 170: 1231–1238. 15911575 10.1534/genetics.105.041087 PMC1451160

[74] Urgaković D, Plohl M (2002) Variation in satellite DNA profiles-causes and effects. EMBO J 21: 5955–5959. 12426367 10.1093/emboj/cdf612 PMC137204

