# Supplementary figures and images for: Molecular Analysis and Genomic Organization of Major DNA Satellites in Banana (Musa spp.)
Source: PLoS One. 2013 Jan 23;8(1):e54808. doi: 10.1371/journal.pone.0054808 (PMC3553004; doi:10.1371/journal.pone.0054808)

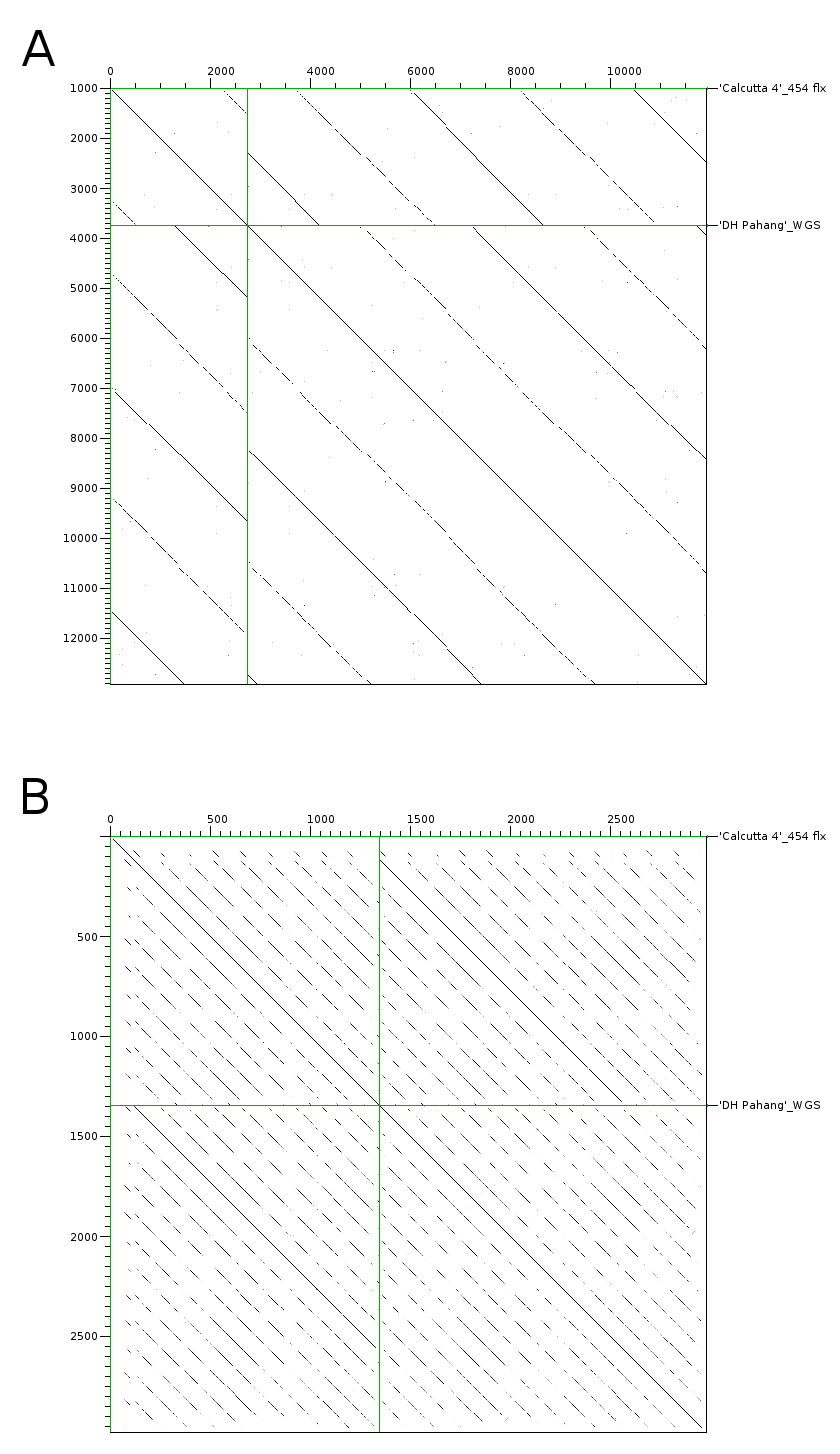

Supplement: Figure S1 — (A) Dot-plot comparison of maTR_CL18 which was identified in 454 sequence data of M. acuminata ‘Calcutta 4’ [13] and CL18-like repeat which was identified in whole genome sequence of M. acuminata ‘DH Pahang’ [35] . Both repetitive units are more than 2.2 kb long and are organized in tandem arrays. (B) Dot-plot comparison of maTR_CL33 which was identified in 454 data of M. acuminata ‘Calcutta 4’ [13] and CL33-like repeat which was identified in whole genome sequence of M. acuminata ‘DH Pahang’ [35]. Both repetitive units are 134 bp long and are organized in tandem arrays. Sequence similarities are represented by dots and diagonal lines (A, B). (TIFF) [file pone.0054808.s001.tiff]

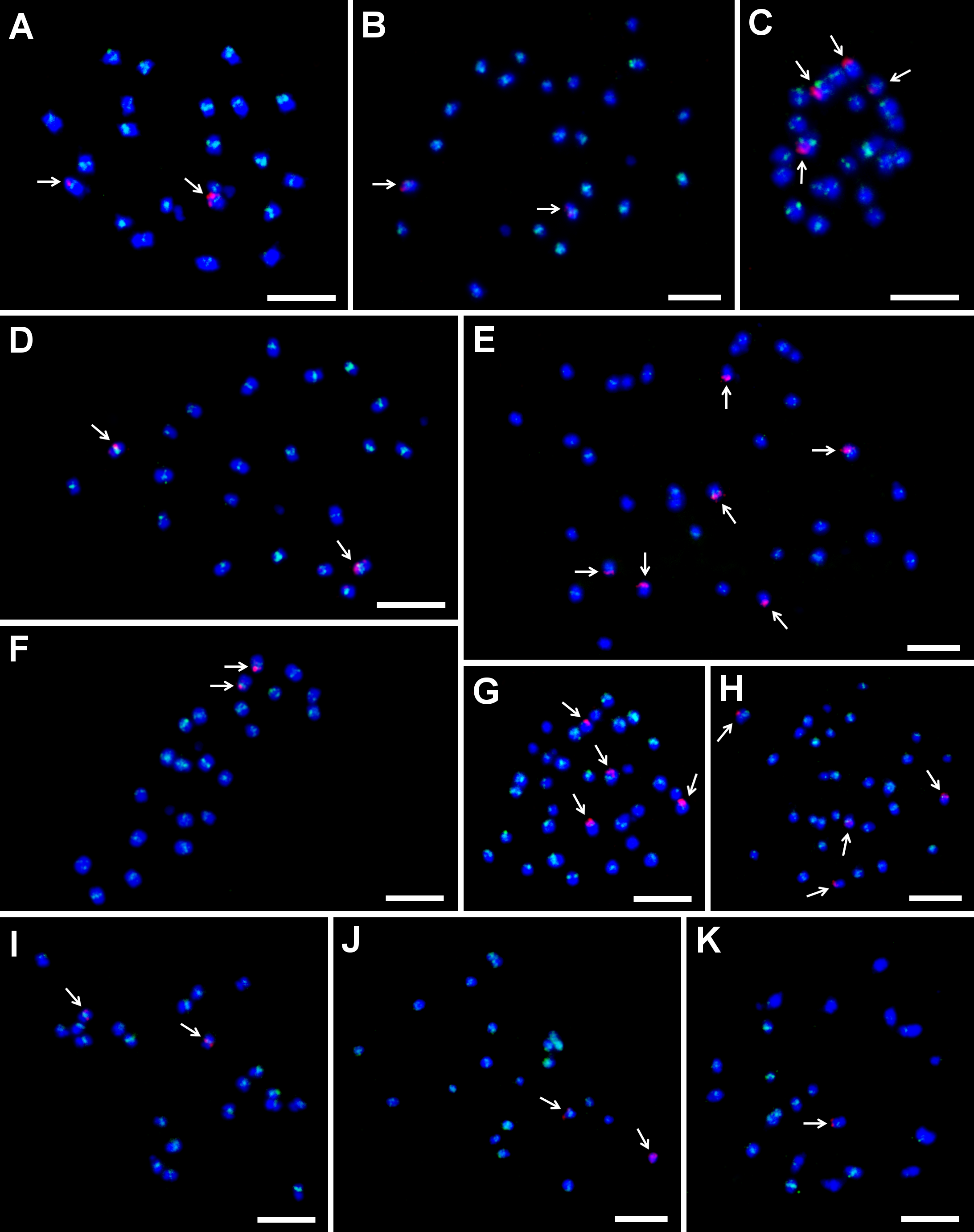

Supplement: Figure S2 — Examples of genomic distribution of satellite DNA as determined on mitotic metaphase chromosomes of Musa after FISH with labeled probes for CL18 (red, labeled by arrows) and banana LINE element (green). Chromosomes were counterstained with DAPI (blue). (A) ‘Maia Oa’. (B) ‘Long Tavoy’. (C) ‘Tani’. (D) M. schizocarpa ITC 0560. (E) ‘Pelipita’. (F) M. schizocarpa ITC 1002. (G) ‘Obino l’Ewai’. (H) ‘Maritú’. (I) ‘Ato’. (J) ‘Tonton Kepa’. (K) ‘Umbubu’. Bar = 5 µm. (TIFF) [file pone.0054808.s002.tiff]

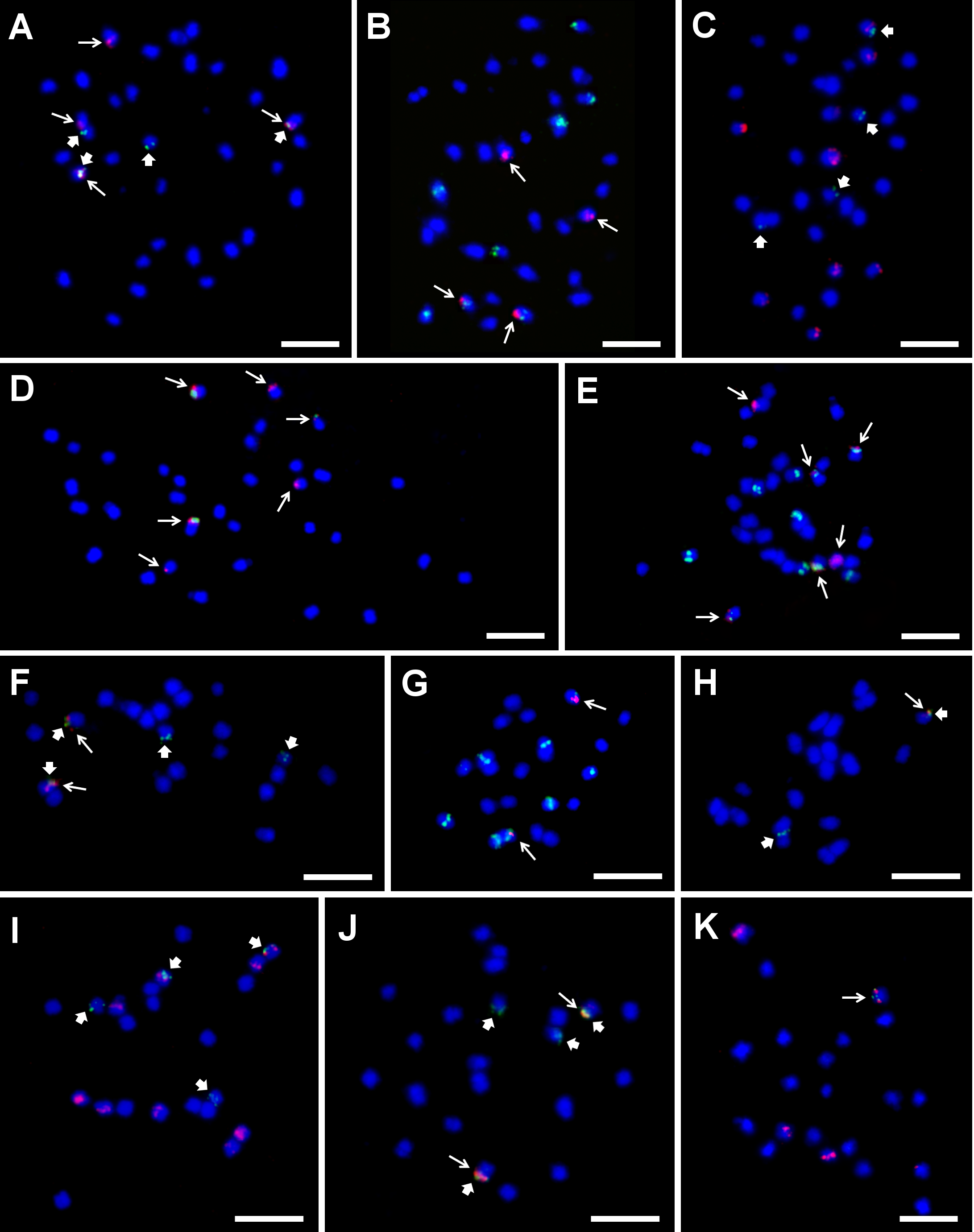

Supplement: Figure S4 — Examples of genomic distribution of satellite DNA as determined on mitotic metaphase chromosomes of interspecific hybrids after FISH. Chromosomes were counterstained with DAPI (blue). Sites of CL18 and CL33 probe hybridization are marked by long and thick arrows, respectively. (A) CL18 (red) and CL33 (green) on chromosomes of ‘Maritú’. (B) CL18 (red) and 5S rDNA (green) on chromosomes of ‘Maritú’. (C) 5S rDNA (red) and CL33 (green) on chromosomes of ‘Maritú’. (D) CL18 (red) and BAC clone 2G17 (green) on chromosomes of ‘Pelipita’. (E) CL18 (red) and 5S rDNA on chromosomes of ‘Pelipita’. (F) CL18 (red) and CL33 (green) on chromosomes of ‘Ato’. (G) CL18 (red) and 5S rDNA (green) on chromosomes of ‘Tonton Kepa’. (H) CL18 (red) and CL33 (green) on chromosomes of ‘Umbubu’. (I) 5S rDNA (red) and CL33 (green) on chromosomes of ‘Ato’. (J) CL18 (red) and CL33 (green) on chromosomes of ‘Tonton Kepa’. (K) 5S rDNA (red) and CL18 (green) on chromosomes of ‘Umbubu’. Bar = 5 µm. (TIFF) [file pone.0054808.s004.tiff]
