# Supplementary material for: Molecular Analysis and Genomic Organization of Major DNA Satellites in Banana (Musa spp.)
Source: PLoS One. 2013 Jan 23;8(1):e54808. doi: 10.1371/journal.pone.0054808 (PMC3553004; doi:10.1371/journal.pone.0054808)
Supplement: Figure S3 — Idiograms of diploid (A – C) and hybrid (D – G) Musa accessions. (A) M. acuminata, (B) M. balbisiana, (C) M. schizocarpa, (D) Hybrids with AAB genomics constitution, (E) Hybrids with ABB genomic constitution, (F) Hybrids with AS genomic constitution, (G) Hybrids with AT genomic constitution. No attempt was made to identify homologs in the hybrids (D – G) and all chromosomes are shown. The chromosome sizes are only indicative. (PDF) [file pone.0054808.s003.pdf]

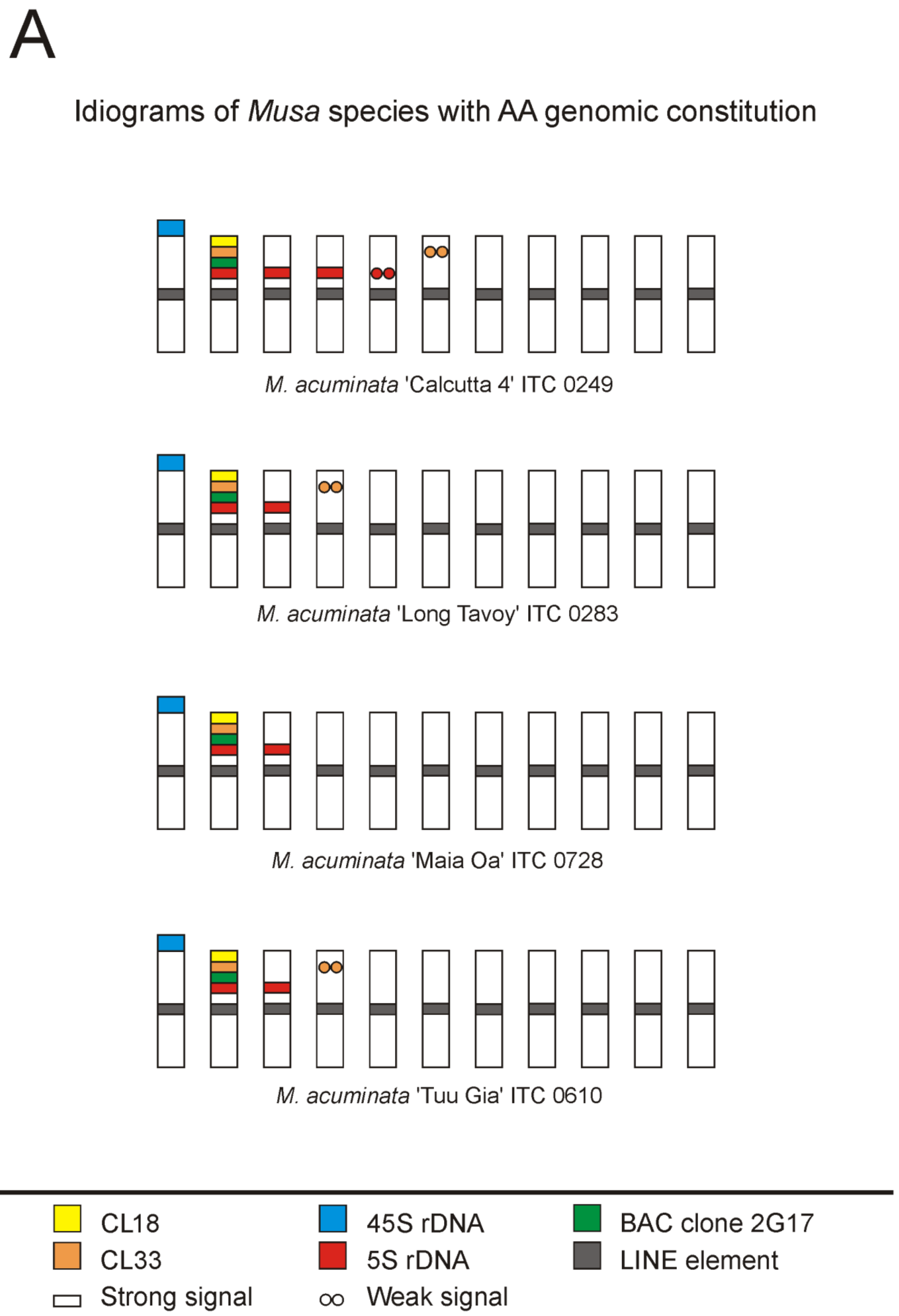
A
Idiograms of Musa species with AA genomic constitution
M. acuminata 'Calcutta 4' ITC 0249
M. acuminata 'Long Tavoy' ITC 0283
M. acuminata 'Maia Oa' ITC 0728
M. acuminata 'Tuu Gia' ITC 0610
CL18
CL33
Strong signal
45S rDNA
5S rDNA
Weak signal
BAC clone 2G17
LINE element

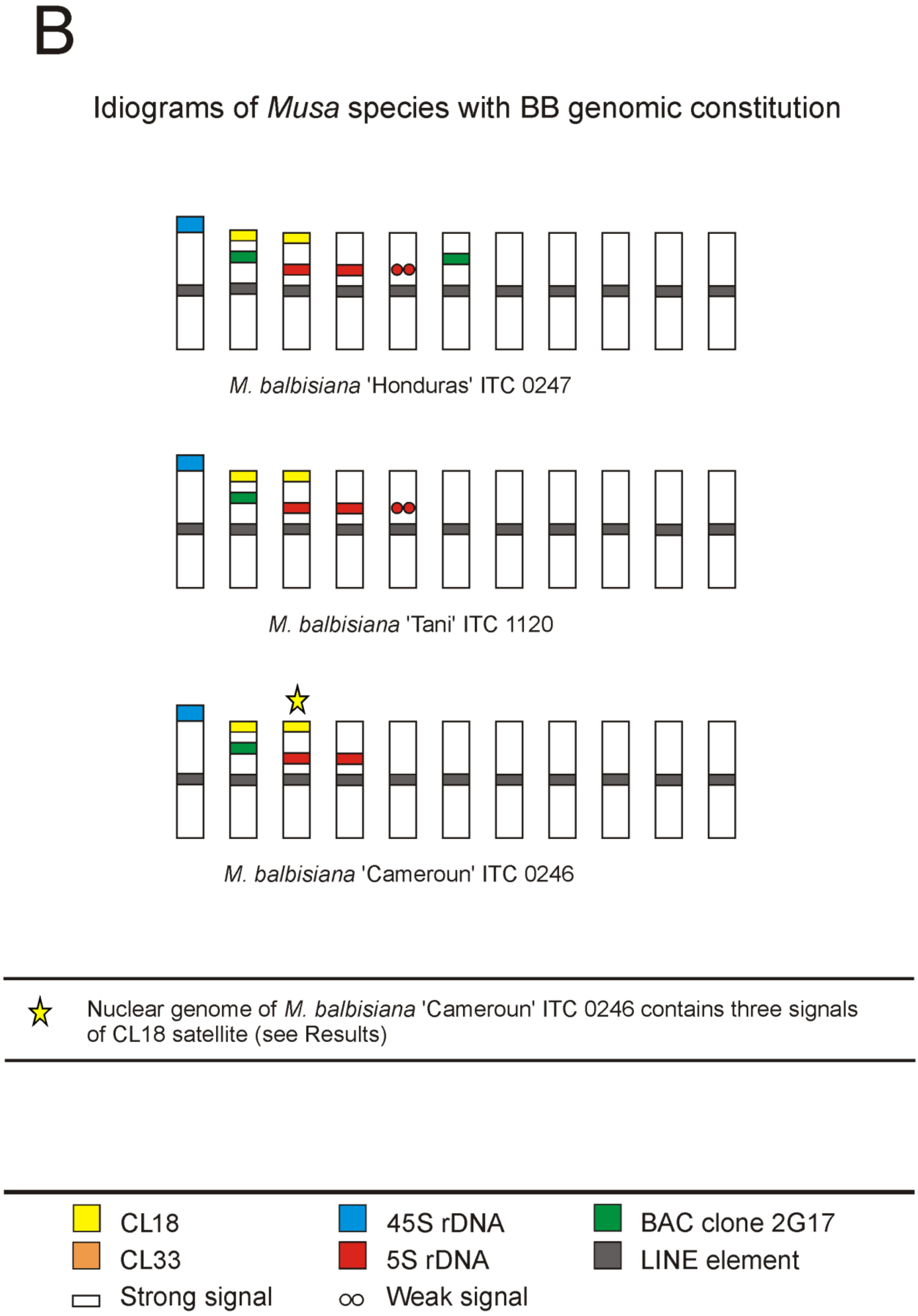
B
Idiograms of Musa species with BB genomic constitution
M. balbisiana 'Honduras' ITC 0247
M. balbisiana 'Tani' ITC 1120
M. balbisiana 'Cameroun' ITC 0246
Nuclear genome of M. balbisiana 'Cameroun' ITC 0246 contains three signals of CL18 satellite (see Results)
CL18
CL33
Strong signal
45S rDNA
5S rDNA
Weak signal
BAC clone 2G17
LINE element

C

Idiogram of *Musa* species with SS genomic constitution

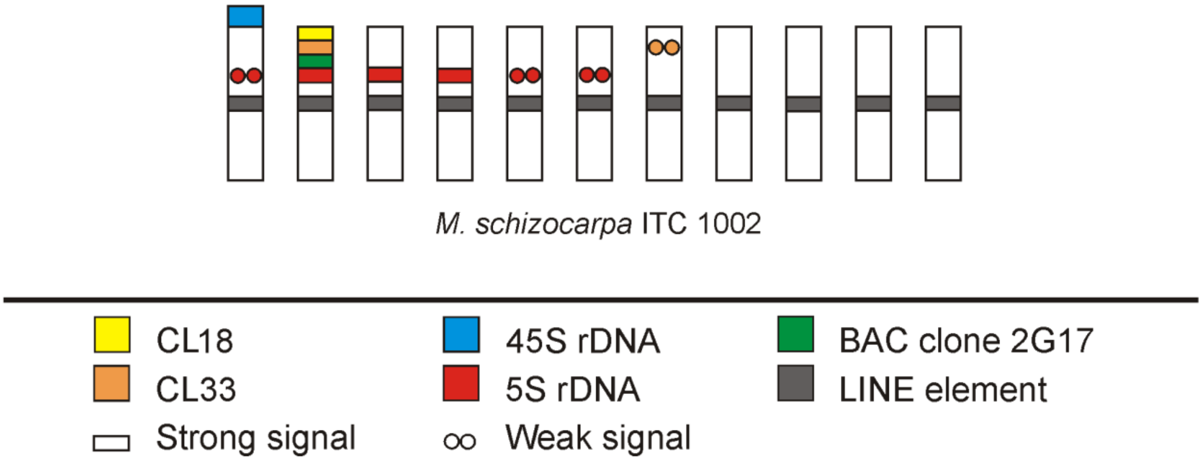

D

Idiograms of *Musa* hybrid clones with AAB genomic constitution

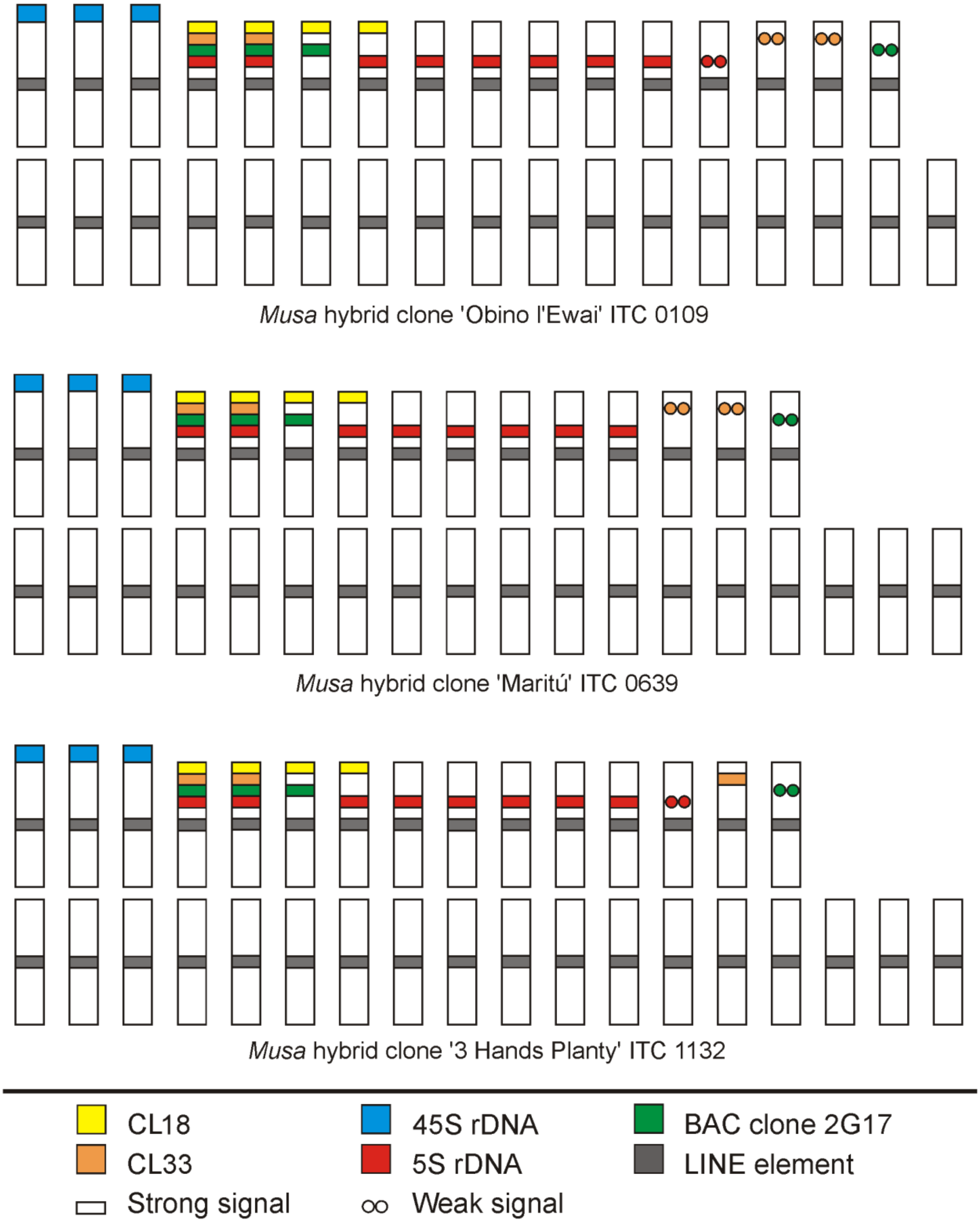

E

Idiograms of *Musa* hybrid clones with ABB genomic constitution

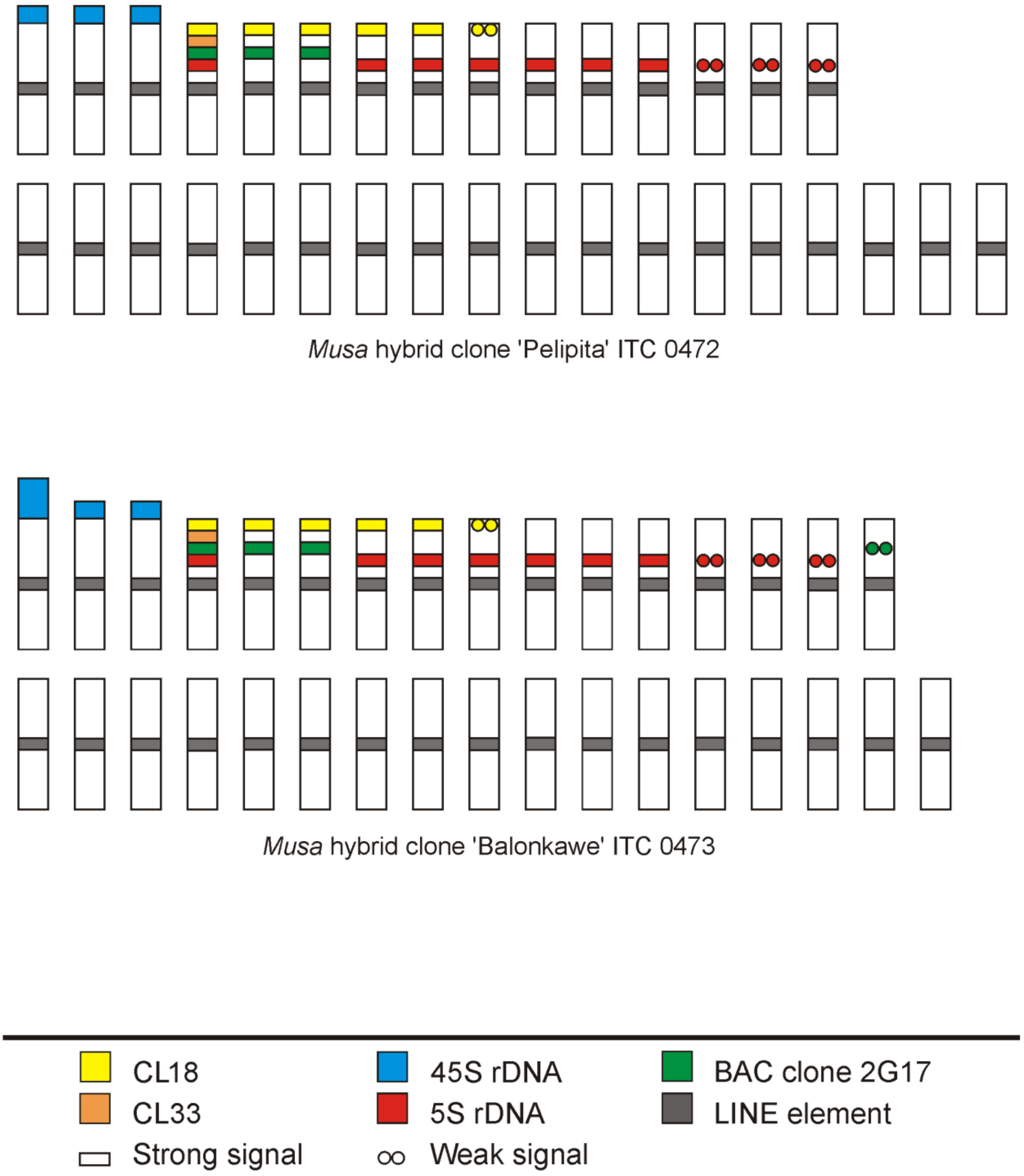

F

Idiograms of *Musa* hybrid clones with AS genomic constitution

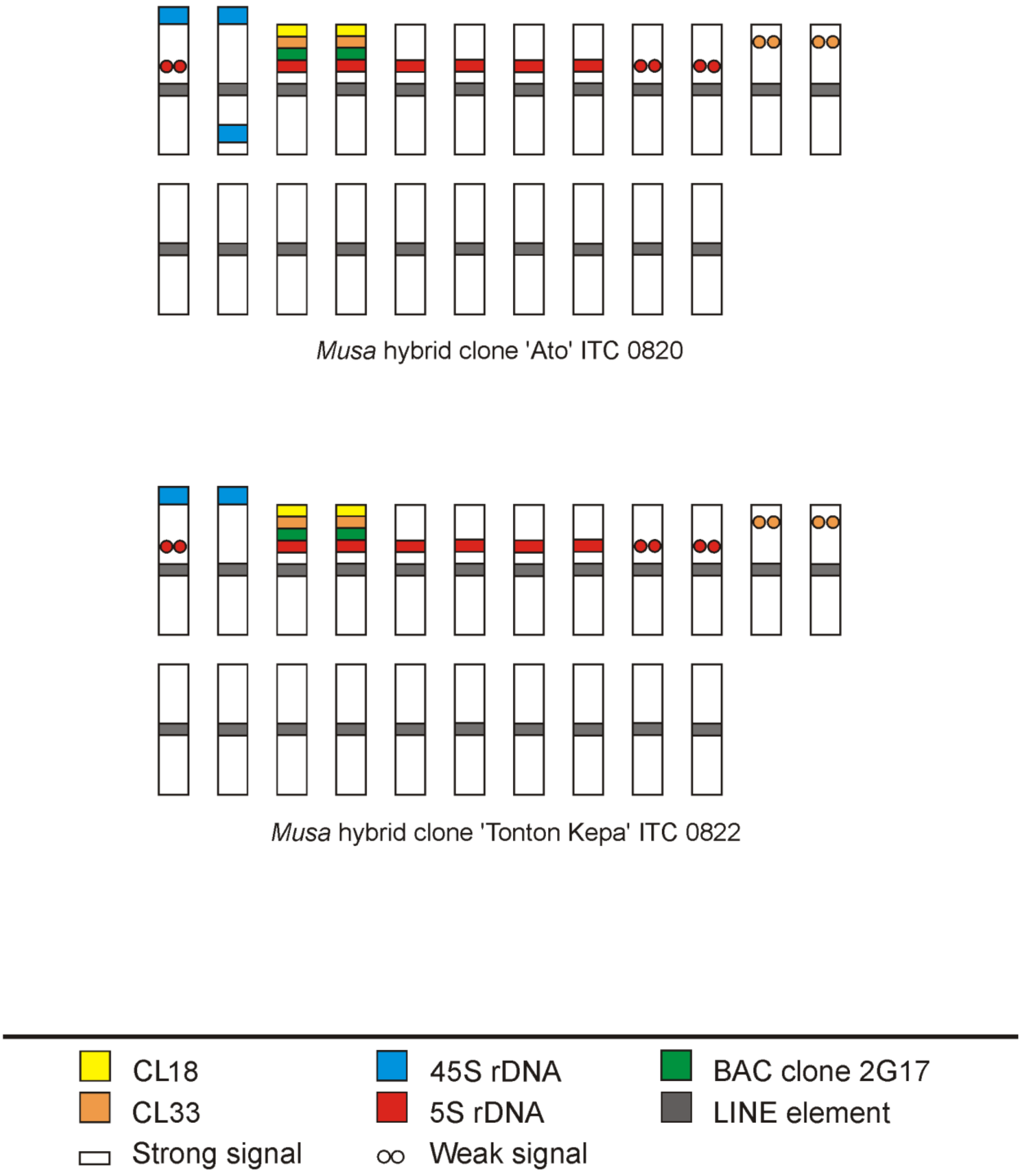

G

Idiogram of *Musa* hybrid clone with AT genomic constitution

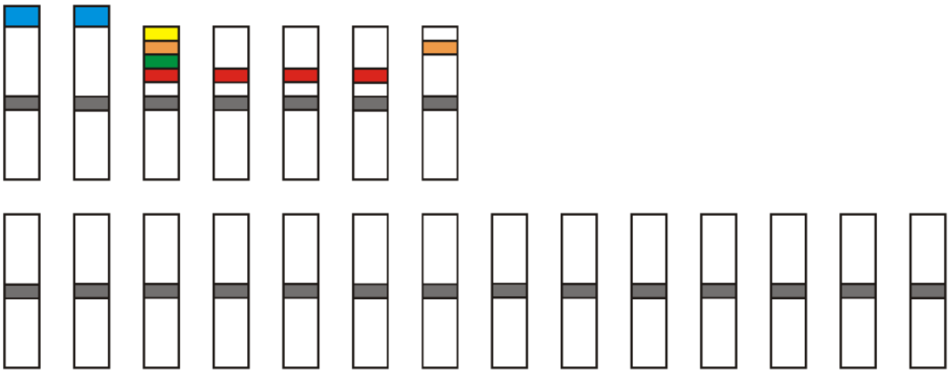

Musa hybrid clone 'Umbubu' ITC 0854

CL18
CL33
Strong signal
45S rDNA
5S rDNA
Weak signal
BAC clone 2G17
LINE element
