# Supplementary material for: Molecular Analysis and Genomic Organization of Major DNA Satellites in Banana (Musa spp.)
Source: PLoS One. 2013 Jan 23;8(1):e54808. doi: 10.1371/journal.pone.0054808 (PMC3553004; doi:10.1371/journal.pone.0054808)
Supplement: Table S1 — Basic characteristics and nucleotide diversity of part1 of CL18-like repeats. (DOC) [file pone.0054808.s005.doc]

**Table S1:** Basic characteristics and nucleotide diversity of part1 of CL18-like repeats

| Accession code (ITC code) |  | Genomic constitution |  | Number of sequenced clones |  | **Note** | |  | Length of sequenced region |  | **Similarity to maTR_CL18 [%]** |  | **Nucleotide diversity** |
| --- | --- | --- | --- | --- | --- | --- | --- | --- | --- | --- | --- | --- | --- |
|  |  |  |  |  |  |  |  |  |  |  |  |  | **θπ** |
|  |  |  |  |  |  |  |  |  |  |  |  |  |  |
| 0249 |  | AA |  | 32 |  |  |  |  | 921 bp |  | 93 – 99 |  | 5.459 |
| 0283 |  | AA |  | 24 |  |  |  |  | 341 bp |  | 95 |  | 2.353 |
| 0728 |  | AA |  | 24 |  |  |  |  | 342 bp |  | 94 |  | 5.987 |
| 1511 |  | AA |  | 24 |  |  |  |  | 342 bp |  | 94 |  | 6.647 |
| 0610 |  | AA |  |  |  | sequences corresponding to part1 were not obtained | |  |  |  |  |  |  |
| 0246 |  | BB |  | 26 |  |  |  |  | 934 bp |  | 94 – 96 |  | 6.171 |
| 0247 |  | BB |  | 24 |  |  |  |  | 344 bp |  | 94 |  | 0.000 |
| 1120 |  | BB |  | 30 |  |  |  |  | 927 bp |  | 94 – 96 |  | 6.451 |
| PKW |  | BB |  | 26 |  |  |  |  | 921 bp |  | 94 – 96 |  | 5.382 |
| 0560 |  | SS |  |  |  | sequences corresponding to part1 were not obtained | |  |  |  |  |  |  |
| 1002 |  | SS |  |  |  | sequences corresponding to part1 were not obtained | |  |  |  |  |  |  |
| 0109 |  | AAB |  | 24 |  |  |  |  | 926 bp |  | 94 – 95 |  | 6.403 |
| 0639 |  | AAB |  | 24 |  |  |  |  | 931 bp |  | 72 – 95 |  | 12.502 |
| 1132 |  | AAB |  | 24 |  |  |  |  | 965 bp |  | 86 – 97 |  | 6.222 |
| 0472 |  | ABB |  | 28 |  |  |  |  | 927 bp |  | 74 – 97 |  | 8.970 |
| 0473 |  | ABB |  | 24 |  |  |  |  | 925 bp |  | 93 – 97 |  | 5.984 |
| 0820 |  | AS |  | 37 |  | Two DNA sequences were obtained | Type 1 |  | 369 bp |  | 59 – 74 |  | 7.256 |
|  |  |  |  |  |  |  | Type 2 |  | 367 bp |  | 50 |  | 5.106 |
| 0822 |  | AS |  | 42 |  |  |  |  | 341 bp |  | 96 |  | 0.185 |
| 0854 |  | AT |  | 24 |  |  |  |  | 924 bp |  | 88 – 96 |  | 4.618 |
|  |  |  |  |  |  |  |  |  |  |  |  |  |  |
