# Supplementary material for: Molecular Analysis and Genomic Organization of Major DNA Satellites in Banana (Musa spp.)
Source: PLoS One. 2013 Jan 23;8(1):e54808. doi: 10.1371/journal.pone.0054808 (PMC3553004; doi:10.1371/journal.pone.0054808)
Supplement: Table S2 — Basic characteristics and nucleotide diversity of part2 of CL18-like repeats. (DOC) [file pone.0054808.s006.doc]

**Table S2:** Basic characteristics and nucleotide diversity of part2 of CL18-like repeats

| Accession code (ITC code) |  | Genomic constitution |  | Number of sequenced clones |  | **Note** | | | |  | Length of sequenced region |  | **Similarity to maTR_CL18 [%]** |  | **Nucleotide diversity** |
| --- | --- | --- | --- | --- | --- | --- | --- | --- | --- | --- | --- | --- | --- | --- | --- |
|  |  |  |  |  |  |  |  |  |  |  |  |  |  |  | **θπ** |
| 0249 |  | AA |  | 24 |  |  |  | |  | | 738 bp |  | 96 -99 |  | 0.667 |
| 0283 |  | AA |  | 27 |  | Two DNA sequences were obtained | | Type 1 |  | | 728 bp |  | 96 |  | 1.818 |
|  |  |  |  |  |  |  |  | Type 2 |  | | 432 bp |  | 74 |  | 0.233 |
| 0728 |  | AA |  | 27 |  |  |  | |  | | 727 bp |  | 95 |  | 0.484 |
| 1511 |  | AA |  | 24 |  |  |  | |  | | 729 bp |  | 95 |  | 0.094 |
| 0610 |  | AA |  | 58 |  | Three DNA sequences were obtained | | Type 1 |  | | 259 bp |  | 61 |  | 0.667 |
|  |  |  |  |  |  |  |  | Type 2 |  | | 361 bp |  | 83 |  | 1.672 |
|  |  |  |  |  |  |  |  | Type 3 |  | | 402 bp |  | 83 |  | 1.181 |
| 0246 |  | BB |  | 42 |  |  |  | |  | | 830 bp |  | 88 – 91 |  | 9.086 |
| 0247 |  | BB |  | 24 |  |  |  | |  | | 823 bp |  | 90 |  | 0.000 |
| 1120 |  | BB |  | 33 |  |  |  | |  | | 746 bp |  | 88 – 94 |  | 8.698 |
| PKW |  | BB |  | 24 |  |  |  | |  | | 693 bp |  | 90 – 95 |  | 0.122 |
| 0560 |  | SS |  | 24 |  |  |  | |  | | 832 bp |  | 66 – 69 |  | 9.232 |
| 1002 |  | SS |  | 30 |  |  |  | |  | | 694 bp |  | 67 – 76 |  | 8.537 |
| 0109 |  | AAB |  | 24 |  |  |  | |  | | 925 bp |  | 70 – 90 |  | 6.403 |
| 0639 |  | AAB |  | 41 |  | Two DNA sequences were obtained | | Type 1 |  | | 670 bp |  | 72 |  | 0.000 |
|  |  |  |  |  |  |  |  | Type 2 |  | | 742 bp |  | 90 |  | 0.210 |
| 1132 |  | AAB |  |  |  | Sequences corresponding to part2 were not obtained | | | |  |  |  |  |  |  |
| 0472 |  | ABB |  | 42 |  |  |  | |  | | 828 bp |  | 88 – 95 |  | 8.555 |
| 0473 |  | ABB |  | 29 |  |  |  | |  | | 767 bp |  | 91 – 95 |  | 1.236 |
| 0820 |  | AS |  | 27 |  |  |  | |  | | 578 bp |  | 83 – 92 |  | 8.075 |
| 0822 |  | AS |  | 35 |  |  |  | |  | | 401 bp |  | 84 |  | 0. 457 |
| 0854 |  | AT |  | 24 |  |  | | | |  | 826 bp |  | 91 – 95 |  | 8.525 |
