# Supplementary material for: Molecular Analysis and Genomic Organization of Major DNA Satellites in Banana (Musa spp.)
Source: PLoS One. 2013 Jan 23;8(1):e54808. doi: 10.1371/journal.pone.0054808 (PMC3553004; doi:10.1371/journal.pone.0054808)
Supplement: Table S3 — Basic characteristics and nucleotide diversity of CL33-like repeats. (DOC) [file pone.0054808.s007.doc]

**Table S3:** Basic characteristics and nucleotide diversity of CL33-like repeats

| Accession code (ITC code) |  | Genomic constitution |  | Number of sequenced clones |  | **Note** | |  | Length of repetitive unit |  | **Similarity to maTR_CL33 [%]** |  | **Nucleotide diversity** |
| --- | --- | --- | --- | --- | --- | --- | --- | --- | --- | --- | --- | --- | --- |
|  |  |  |  |  |  |  |  |  |  |  |  |  | **θπ** |
| 0249 |  | AA |  | 61 |  |  |  |  | 134 bp |  | 99 |  | 3.500 |
| 0283 |  | AA |  | 26 |  | Two DNA sequences were obtained | Type 1 |  | 134 bp |  | 93 – 98 |  | 3.194 |
|  |  |  |  |  |  |  | Type 2 |  | 110 bp |  |  |  | 0.000 |
| 0728 |  | AA |  | 36 |  | Two DNA sequences were obtained | Type 1 |  | 134 bp |  | 97 |  | 2.667 |
|  |  |  |  |  |  |  | Type 2 |  | 188 bp |  |  |  | 0.692 |
| 1511 |  | AA |  | 24 |  |  |  |  | 134 bp |  | 98 – 99 |  | 4.248 |
| 0610 |  | AA |  | 24 |  |  |  |  | 188 bp |  |  |  | 0.500 |
| 0246 |  | BB |  | 24 |  |  |  |  | 134 bp |  | 96 – 98 |  | 3.225 |
| 0247 |  | BB |  | 24 |  |  |  |  | 134 bp |  | 87 – 98 |  | 13.285 |
| 1120 |  | BB |  | 24 |  |  |  |  | 134 bp |  | 86 – 97 |  | 4.945 |
| PKW |  | BB |  | 27 |  |  |  |  | 134 bp |  | 93 – 98 |  | 5.703 |
| 0560 |  | SS |  | 31 |  |  |  |  | 134 bp |  | 84 |  | 1.810 |
| 1002 |  | SS |  | 24 |  |  |  |  | 134 bp |  | 83 – 86 |  | 4.862 |
| 0109 |  | AAB |  | 39 |  |  |  |  | 134 bp |  | 95 – 98 |  | 4.825 |
| 0639 |  | AAB |  | 38 |  |  |  |  | 134 bp |  | 85 – 98 |  | 9.387 |
| 1132 |  | AAB |  | 30 |  | Two DNA sequences were obtained | Type 1 |  | 134 bp |  | 87 – 96 |  | 4.713 |
|  |  |  |  |  |  |  | Type 2 |  | 188 bp |  |  |  | 0.667 |
| 0472 |  | ABB |  | 33 |  | Two DNA sequences were obtained | Type 1 |  | 134 bp |  | 86 – 97 |  | 5.464 |
|  |  |  |  |  |  |  | Type 2 |  | 188 bp |  |  |  | 0.250 |
| 0473 |  | ABB |  | 33 |  |  |  |  | 134 bp |  | 84 – 100 |  | 15.696 |
| 0820 |  | AS |  | 24 |  | Two DNA sequences were obtained | Type 1 |  | 134 bp |  | 84 – 98 |  | 6.765 |
|  |  |  |  |  |  |  | Type 2 |  | 188 bp |  |  |  | 0.400 |
| 0822 |  | AS |  | 28 |  | Two DNA sequences were obtained | Type 1 |  | 134 bp |  | 83 – 97 |  | 6.633 |
|  |  |  |  |  |  |  | Type 2 |  | 188 bp |  |  |  | 0.750 |
| 0854 |  | AT |  | 41 |  |  |  |  | 134 bp |  | 83 – 95 |  | 4.265 |
