# Supplementary material for: Molecular Analysis and Genomic Organization of Major DNA Satellites in Banana (Musa spp.)
Source: PLoS One. 2013 Jan 23;8(1):e54808. doi: 10.1371/journal.pone.0054808 (PMC3553004; doi:10.1371/journal.pone.0054808)
Supplement: Table S4 — Copy numbers of maTR_CL18 and maTR_CL33 satellites. (DOC) [file pone.0054808.s008.doc]

**Table S4:** Copy numbers of maTR_CL18 and maTR_CL33 satellites

| **Species** |  | Subspecies |  | **Accession name** |  | **ITC code** |  | **maTR_CL18** | | |  | **maTR_CL33** | | | | |
| --- | --- | --- | --- | --- | --- | --- | --- | --- | --- | --- | --- | --- | --- | --- | --- | --- |
|  |  |  |  |  |  |  |  | Copy number/1C_x_ |  | Genome proportion [%] |  | Copy number/1Cx_x_ |  | Genome proportion [%] | | |
|  |  |  |  |  |  |  |  |  |  |  |  |  |  |  | | |
| *M. acuminata* |  |  |  |  |  |  |  |  |  |  |  |  |  |  | | |
|  |  | *burmannicoides* |  | Calcutta4 |  | 0249 |  | ~ 1x10^3^ - 2x10^3^ |  | 0.39 – 0.73 |  | ~ 5.5x10^3^ - 7x10^3^ |  | 0.12 – 0.16 | | |
|  |  | *burmannica* |  | Long Tavoy |  | 0283 |  | ~ 1x10^3^ - 2x10^3^ |  | 0.39 – 0.73 |  | ~ 2.5x10^3^ - 4x10^3^ |  | 0.06 – 0.09 | | |
|  |  | *zebrina* |  | Maia Oa |  | 0728 |  | ~ 1x10^3^ - 2x10^3^ |  | 0.34 – 0.68 |  | ~ 2.5x10^3^ - 4x10^3^ |  | 0.05 – 0.08 | | |
|  |  | *malaccensis* |  | DH Pahang |  | 1511 |  | ~ 3.5x10^3^ - 5x10^3^ |  | 1.29 – 1.85 |  | ~ 5.5x10^3^ - 7x10^3^ |  | 0.12 – 0.16 | | |
|  |  |  |  | Tuu Gia |  | 0610 |  | ~ 1x10^3^ - 2x10^3^ |  | 0.36 – 0.71 |  | ~ 2.5x10^3^ - 4x10^3^ |  | 0.05 – 0.09 | | |
|  |  |  |  |  |  |  |  |  |  |  |  |  |  |  | | |
| *M. balbisiana* |  |  |  |  |  |  |  |  |  |  |  |  |  |  | | |
|  |  |  |  | Cameroun |  | 0246 |  | ~ 3.5x10^3^ - 5x10^3^ |  | 1.41 – 2.01 |  | below detection limit |  | --- | | |
|  |  |  |  | Honduras |  | 0247 |  | ~ 1x10^3^ - 2x10^3^ |  | 0.40 – 0.79 |  | below detection limit |  | --- | | |
|  |  |  |  | Tani |  | 1120 |  | ~ 3.5x10^3^ - 5x10^3^ |  | 1.40 – 2.00 |  | below detection limit |  | --- | | |
|  |  |  |  | Pisang Klutuk Wulung |  | --- |  | ~ 3.5x10^3^ - 5x10^3^ |  | 1.39 – 1.99 |  | below detection limit |  | --- | | |
|  |  |  |  |  |  |  |  |  |  |  |  |  |  |  | | |
| *M. schizocarpa* |  |  |  |  |  |  |  |  |  |  |  |  |  |  | | |
|  |  |  |  | *Musa schizocarpa* |  | 0560 |  | ~ 1x10^3^ - 2x10^3^ |  | 0.33 – 0.66 |  | ~ 5.5x10^3^ - 7x10^3^ |  | 0.11 – 0.14 | | |
|  |  |  |  | *Musa schizocarpa* |  | 1002 |  | ~ 1x10^3^ - 2x10^3^ |  | 0.33 – 0.66 |  | ~ 5.5x10^3^ - 7x10^3^ |  | 0.11 – 0.14 | | |
|  |  |  |  |  |  |  |  |  |  |  |  |  |  |  | | |
| Hybrids |  |  |  |  |  |  |  |  |  |  |  |  |  |  | | |
|  |  |  |  | Obino l'Ewai |  | 0109 |  | ~ 5x10^3^ - 6.5x10^3^ |  | 1.90 – 2.47 |  | ~ 5.5x10^3^ - 7x10^3^ |  | 0.13 – 0.16 | | |
|  |  |  |  | Maritú |  | 0639 |  | ~ 3x10^3^ - 4x10^3^ |  | 1.10 – 1.46 |  | ~ 5.5x10^3^ - 7x10^3^ |  | 0.12 – 0.16 | | |
|  |  |  |  | 3 Hands Planty |  | 1132 |  | ~ 3x10^3^ - 4x10^3^ |  | 1.13 – 1.51 |  | ~ 5.5x10^3^ - 7x10^3^ |  | 0.13 – 0.16 | | |
|  |  |  |  | Pelipita |  | 0472 |  | ~ 5x10^3^ - 6.5x10^3^ |  | 1.93 – 2.50 |  | below detection limit |  | --- | | |
|  |  |  |  | Balonkawe |  | 0473 |  | ~ 5x10^3^ - 6.5x10^3^ |  | 1.86 – 2.42 |  | below detection limit |  | --- | | |
|  |  |  |  | Ato |  | 0820 |  | ~ 1x10^3^ - 2x10^3^ |  | 0.35 – 0.70 |  | ~ 2.5x10^3^ - 4x10^3^ |  | 0.05 – 0.08 | | |
|  |  |  |  | Tonton Kepa |  | 0822 |  | ~ 1x10^3^ - 2x10^3^ |  | 0.34 – 0.68 |  | ~ 2.5x10^3^ - 4x10^3^ |  | 0.05 – 0.08 | | |
|  |  |  |  | Umbubu |  | 0854 |  | ~ 3x10^3^ - 4x10^3^ |  | 0.97 – 1.36 |  | ~ 5.5x10^3^ - 7x10^3^ |  | 0.11 – 0.14 | | |
|  |  |  |  |  |  |  |  |  |  |  |  |  | | |  |  |
